# A Pathway-Driven Computational Drug Discovery Framework Integrating Machine Learning and Virtual Screening for Identification of Anti-Rheumatoid Arthritis Natural Small Molecules

**DOI:** 10.3390/ph19101651

**Published:** 2026-10-19

**Authors:** Jijia Sun, Yuezhen Wang, Hong Ye, Jialiang Chen, Yilin Chen, Guangtao Yao

**Affiliations:** 1 Laboratory of Mathematical and Computational Intelligence, School of Artificial Intelligence, Shanghai University of Traditional Chinese Medicine, Shanghai 201203, China; jijiasun@163.com (J.S.);; 2 Center for Drug Safety Evaluation, Institute of Innovative Chinese Medicine, Shanghai University of Traditional Chinese Medicine, Shanghai 201203, China

**Keywords:** rheumatoid arthritis, ensemble learning, pathway prioritization, C3AR1, virtual screening, molecular dynamics, natural products

## Abstract

**Objective:** This study aimed to establish a pathway-driven computational drug discovery framework integrating machine learning, target prioritization, virtual screening, molecular dynamics (MD) simulations, and in vitro evaluation to identify natural small molecules with potential anti-rheumatoid arthritis (RA) activity. **Methods:** Sixteen RA-related Gene Expression Omnibus (GEO) datasets were included, with 15 cohorts used for model development and one reserved for independent external validation. Pathway features quantified by gene set variation analysis (GSVA) were subjected to cohort-aware feature selection using a leave-one-dataset-out framework across seven machine-learning algorithms. Predictive modeling and SHapley Additive exPlanations (SHAP) analysis were subsequently performed, followed by random-effects meta-analysis (REMA) and network analysis for candidate-target prioritization. Natural compounds were screened by molecular docking and further evaluated using ligand efficiency, multiple scoring approaches, explicit-membrane MD simulations, and a TNF-α-induced MH7A inflammatory model. **Results:** Ten stable RA-associated pathways were identified. The final CAT+ET model achieved ROC-AUCs of 0.783 (95% CI, 0.731–0.836) and 0.610 (95% CI, 0.496–0.720) in the internal and independent external validation sets, respectively, indicating limited cross-cohort generalizability. SHAP and cross-cohort analyses supported Complement and coagulation cascades for downstream investigation, and integrated expression and network evidence prioritized C3AR1 as a candidate target. Virtual screening identified four candidate natural compounds (SM1–SM4). Ligand-size analysis, ligand efficiency, multiple scoring approaches, and explicit-membrane MD simulations provided complementary structural and energetic assessments of these candidates. Among them, SM1 showed comparatively favorable computational characteristics and a relatively consistent reduction in the inflammatory markers IL6 and MMP3 in TNF-α-stimulated MH7A cells, accompanied by changes in *C3AR1* expression. These convergent computational and experimental findings supported the prioritization of SM1 for further investigation. **Conclusions:** This study established a pathway-driven framework linking cross-cohort pathway identification with candidate-target prioritization and natural-product screening in RA. Complement and coagulation cascades and C3AR1 were prioritized for downstream investigation, while integrated computational and experimental evidence identified SM1 as a candidate with promising anti-inflammatory potential. Although direct compound–C3AR1 engagement and its functional involvement require further validation, the present study provides a systematic and extensible strategy for translating robust RA-associated pathway signals into candidate targets and natural compounds, offering a valuable foundation for subsequent natural-product drug discovery in RA.

## 1. Introduction

Rheumatoid arthritis (RA) is a systemic autoimmune disorder characterized primarily by chronic inflammation of synovial joints and potential involvement of multiple organ systems [1]. The pathological features of RA include inflammatory cell infiltration within the synovium, synovial hyperplasia, pannus formation, and vascular inflammation, which subsequently lead to articular cartilage erosion, bone destruction, and irreversible structural damage of joints [2]. These pathological changes ultimately result in joint deformity, functional impairment, and even disability, severely compromising patients’ quality of life [3]. Beyond articular manifestations, RA may also affect extra-articular organs, including the lungs, lacrimal glands, salivary glands, and cardiovascular system, leading to complications such as interstitial lung disease, secondary Sjögren’s syndrome, and cardiovascular disorders, thereby further increasing disease burden and mortality risk [4,5].

Currently, clinical management of RA mainly involves non-steroidal anti-inflammatory drugs (NSAIDs), glucocorticoids, conventional synthetic disease-modifying antirheumatic drugs (csDMARDs), biological agents, and targeted synthetic disease-modifying antirheumatic drugs (tsDMARDs) [6,7]. Although existing therapeutic strategies can effectively alleviate inflammatory responses, control disease activity, and delay structural joint damage, several limitations remain, including substantial heterogeneity in therapeutic responses, long-term treatment-associated adverse effects (such as increased infection risk and hepatotoxicity/nephrotoxicity), and considerable economic burden. Particularly in advanced-stage disease accompanied by cartilage degeneration and bone erosion, current therapies exhibit limited capacity to promote joint tissue repair and functional restoration. Therefore, developing innovative therapeutic strategies that simultaneously achieve high efficacy, safety, and accessibility, while enabling coordinated regulation of inflammation and tissue repair, remains a critical challenge in precision medicine for RA.

The initiation and progression of RA involve multiple pathological processes, including immune dysregulation, excessive release of inflammatory mediators, activation of fibroblast-like synoviocytes (FLSs), enhanced osteoclast differentiation, and degradation of cartilage extracellular matrix. These processes are fundamentally governed by complex molecular networks and aberrant signaling pathway regulation [8,9,10]. Previous studies have demonstrated that several signaling pathways, including NF-κB [11], JAK-STAT [12], PI3K-Akt [13], MAPK [14], TNF [15], IL-17 [16], and Toll-like receptor signaling pathways [17], play crucial roles in inflammatory amplification, synovial invasion, and bone destruction during RA progression. These pathways not only provide insights into the potential mechanisms underlying RA pathogenesis but also serve as important theoretical foundations for the development of targeted therapeutic agents. Therefore, systematic characterization of RA-associated pathway alterations at the pathway level may facilitate the identification of biologically informative and therapeutically actionable targets.

In recent years, advances in high-throughput transcriptomics, bioinformatics, and machine learning have enabled systematic investigation of disease-associated molecular alterations using publicly available datasets [18,19,20]. Among these resources, the Gene Expression Omnibus (GEO) contains extensive RA-related transcriptomic datasets that provide opportunities for identifying reproducible molecular features across independent cohorts [21]. However, substantial heterogeneity in detection platforms, tissue sources, patient populations, and study design may limit the reproducibility and generalizability of findings derived from individual datasets or conventional sample-level validation strategies. Moreover, most transcriptomic studies have focused primarily on individual genes, whereas pathway-level features may provide a more integrated representation of coordinated biological alterations. Therefore, analytical strategies that explicitly account for inter-cohort heterogeneity while identifying stable pathway-level features may improve the robustness and biological interpretability of RA-associated molecular signatures [22].

Natural products have attracted considerable attention in autoimmune disease drug discovery due to their structural diversity, extensive biological sources, multi-target regulatory properties, and favorable safety profiles [23,24]. However, conventional natural product screening approaches largely depend on empirical knowledge and repeated experimental validation, resulting in prolonged development cycles, high costs, and limited screening efficiency. Recently, advances in bioinformatics, artificial intelligence, and computational drug discovery technologies have provided powerful tools for efficient screening and mechanistic investigation of natural products. Therefore, integrating computational approaches, including key pathway analysis, machine learning, molecular docking, and molecular dynamics (MD) simulation, may enable rapid identification of promising therapeutic compounds from large-scale natural product libraries and provide novel strategies for natural product-based drug discovery and target optimization.

**Figure 1:**
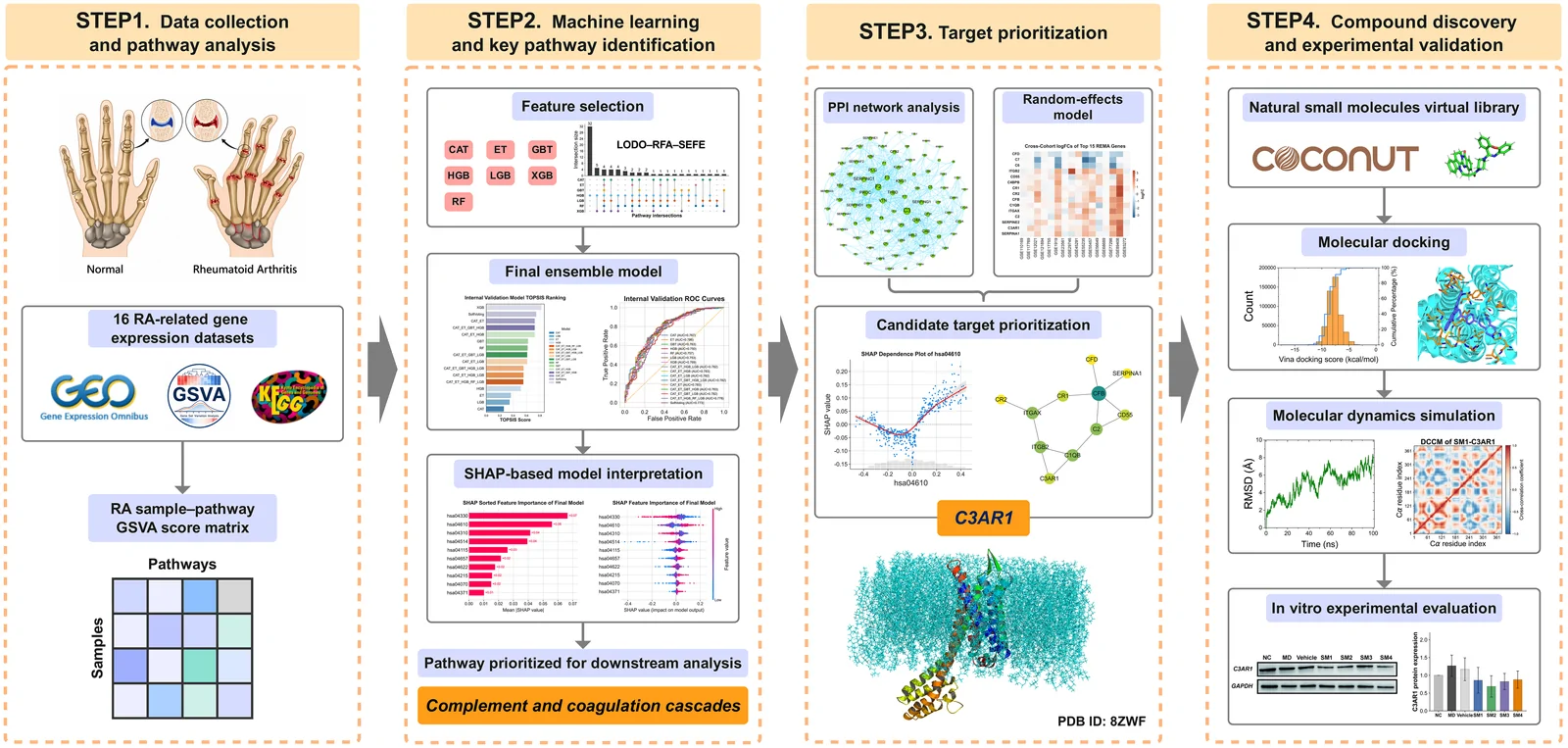
Overall workflow of the pathway-driven computational drug discovery framework.

In this study, we developed a pathway-driven computational drug discovery framework integrating multi-cohort transcriptomic analysis, ensemble learning, candidate-target prioritization, natural-product virtual screening, MD simulations, and in vitro evaluation. RA-associated pathway features were identified using a cohort-aware feature-selection strategy and evaluated across independent datasets, followed by model interpretation to prioritize pathways for downstream investigation. Candidate targets within the selected pathway were subsequently evaluated by integrating cross-cohort expression evidence with network-level information. A large-scale natural-product library was then screened using structure-based approaches, and selected compounds were further characterized by molecular docking and explicit-membrane MD simulations. Finally, their anti-inflammatory effects and associated molecular-expression changes were evaluated in a TNF-α-induced MH7A fibroblast-like synoviocyte model. Through this multilevel framework, we aimed to connect robust pathway-level disease features with candidate targets and natural small molecules for further investigation in RA. The overall workflow is illustrated in Figure 1.

## 2. Results

### 2.1. Identification of Robust RA-Associated Pathway Features Using the LODO–RFA–SEFE Framework

In this study, RA-related gene expression profiling datasets were retrieved from the GEO database using “Rheumatoid Arthritis” as the search keyword. Datasets were further screened according to the inclusion criterion that both RA patient samples and healthy control samples were available. Finally, 16 eligible datasets were included in this study, and detailed information is provided in Table 1.

Based on literature review and systematic analysis, 77 RA-associated pathways were identified from a total of 359 Kyoto Encyclopedia of Genes and Genomes (KEGG) pathways. Subsequently, gene set variation analysis (GSVA) was performed on 16 RA-related gene expression profiling datasets, and the resulting pathway enrichment scores were used as the input dataset for key pathway feature selection. The development dataset consisted of 1498 samples, including 1191 RA samples and 307 healthy controls.

To minimize the influence of cohort-specific characteristics and model-dependent biases on pathway feature selection, we performed cohort-aware pathway screening using a leave-one-dataset-out (LODO) framework combined with recursive feature addition (RFA) and stepwise exhaustive feature elimination (SEFE) across 15 development GEO cohorts. Seven ensemble learning algorithms, including CatBoost (CAT), extremely randomized trees (ET), gradient boosting tree (GBT), histogram-based gradient boosting (HGB), Light Gradient Boosting Machine (LGB), random forest (RF), and Extreme Gradient Boosting (XGB), were independently evaluated for their ability to identify stable pathway features.

**Figure 2:**
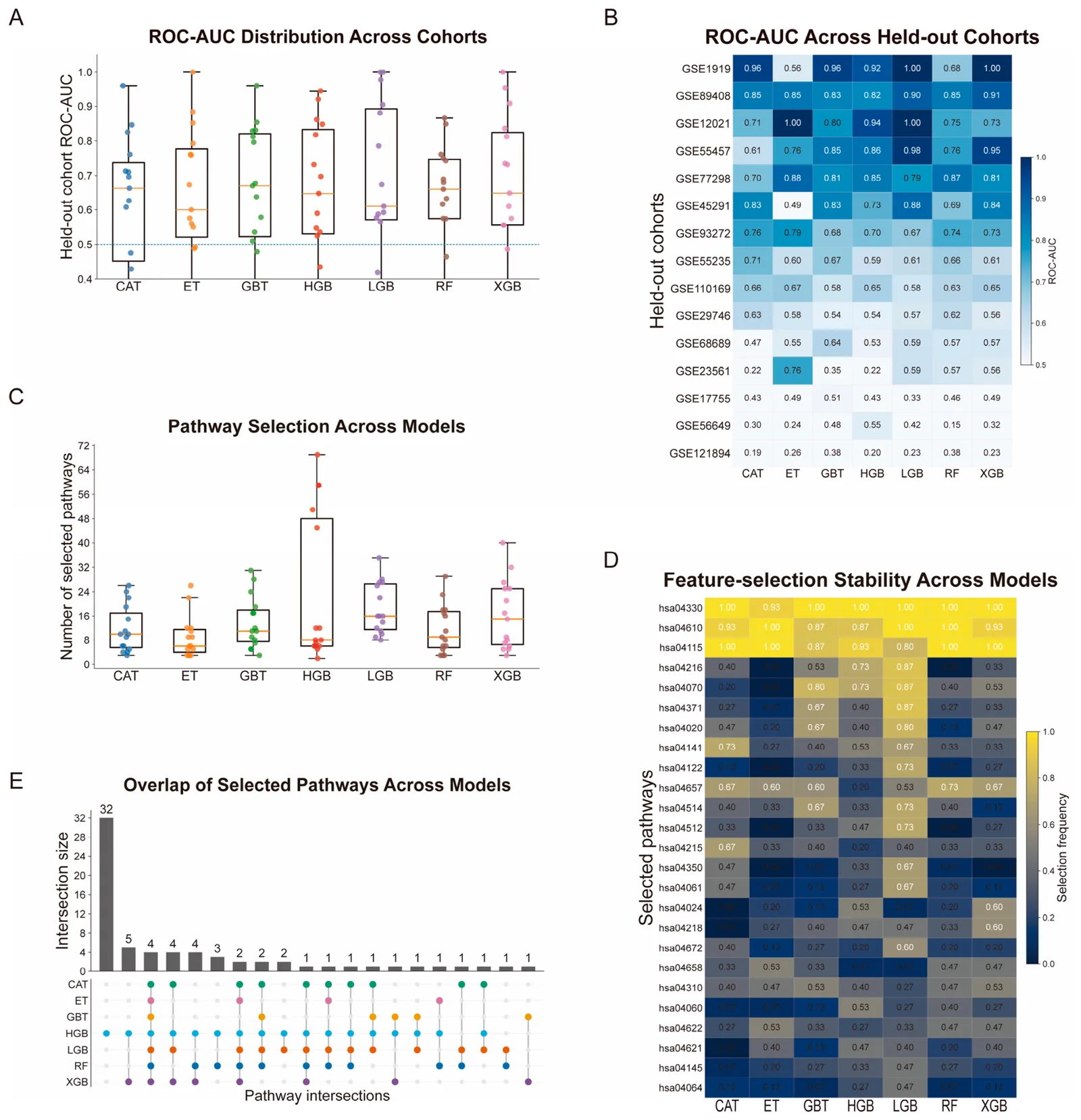
Cohort-aware pathway feature selection using the LODO–RFA–SEFE framework. (**A**) Distribution of ROC-AUC values obtained for the seven learning algorithms across held-out GEO cohorts during leave-one-dataset-out (LODO) evaluation. Each point represents the ROC-AUC obtained when one complete GEO cohort was held out for evaluation. (**B**) Heatmap showing cohort-specific ROC-AUC values for each of the seven learning algorithms across the 15 held-out GEO cohorts. (**C**) Distribution of the number of pathways retained by each learning algorithm across LODO iterations, illustrating variation in feature-set size across algorithms and held-out cohorts. (**D**) Heatmap showing the cross-cohort selection frequency of pathways across the seven learning algorithms. Selection frequency was defined as the proportion of LODO folds in which a given pathway was retained by the corresponding algorithm. (**E**) UpSet plot showing the overlap of pathways selected by the seven learning algorithms after application of the RFA–SEFE feature-selection procedure.

The distribution of held-out cohort area under the receiver operating characteristic curve (ROC-AUC) values across the seven machine learning algorithms is shown in Figure 2A. Although the predictive performance varied among different algorithms, all models demonstrated the ability to distinguish RA samples from controls across multiple held-out cohorts, while the variation in performance across cohorts indicated the presence of inter-cohort heterogeneity. Detailed cohort-level performance evaluation is presented in Figure 2B, where ROC-AUC values were calculated independently for each held-out GEO cohort and each machine learning algorithm. These results demonstrate the necessity of cohort-level validation rather than random sample-level partitioning for evaluating the robustness of pathway-based predictive features.

The number of pathways retained by each machine learning algorithm across LODO iterations is summarized in Figure 2C. Different algorithms selected variable numbers of pathway features, suggesting that individual learning algorithms may capture partially distinct pathway patterns. Therefore, pathway robustness was further evaluated from two complementary perspectives: cross-cohort selection stability and cross-model consensus.

The cross-cohort stability of pathway selection across different machine learning algorithms is illustrated in Figure 2D. Several pathways exhibited consistently high selection frequencies across LODO iterations and learning algorithms. Among them, the Notch signaling pathway (hsa04330), Complement and coagulation cascades (hsa04610), and p53 signaling pathway (hsa04115) showed particularly high selection stability. Specifically, hsa04330 exhibited selection frequencies of 1.00, 0.93, 1.00, 1.00, 1.00, 1.00, and 1.00 across CAT, ET, GBT, HGB, LGB, RF, and XGB, respectively. Similarly, hsa04610 showed selection frequencies of 0.93, 1.00, 0.87, 0.87, 1.00, 1.00, and 0.93, whereas hsa04115 showed frequencies of 1.00, 1.00, 0.87, 0.93, 0.80, 1.00, and 1.00 across the seven algorithms. These findings indicate that these pathways were repeatedly identified under different cohort-held-out conditions and algorithmic frameworks, supporting their robustness as pathway-level predictive features.

Because different machine learning algorithms may identify partially overlapping but non-identical pathway sets, we further integrated the results from all seven models using a consensus-based selection strategy. Specifically, only pathways identified by at least five of the seven machine learning algorithms were retained as the final pathway feature set. The overlap among pathway features selected by different algorithms is shown in Figure 2E. Based on this predefined consensus criterion, a total of 10 robust pathway features were identified, including Wnt signaling pathway (hsa04310), Notch signaling pathway (hsa04330), Complement and coagulation cascades (hsa04610), IL-17 signaling pathway (hsa04657), p53 signaling pathway (hsa04115), RIG-I-like receptor signaling pathway (hsa04622), Phosphatidylinositol signaling system (hsa04070), Apoptosis–multiple species (hsa04215), Apelin signaling pathway (hsa04371), and Cell adhesion molecules (hsa04514).

These consensus pathways were subsequently used as fixed pathway features for downstream predictive model development and evaluation. Overall, the LODO-based analysis demonstrated that pathway selection exhibited certain algorithm- and cohort-dependent variability; however, integrating repeated cross-cohort selection patterns with cross-model consensus enabled the identification of a robust set of RA-associated pathway features for subsequent machine-learning modeling and biological prioritization.

### 2.2. Construction and Validation of the Ensemble Predictive Model Based on Key Pathway Features

Based on the 10 key pathway features identified through the LODO–RFA–SEFE feature-selection framework and cross-model consensus analysis, we subsequently reconstructed the predictive models for RA classification. Seven base classifiers were first trained using the fixed pathway feature set, and Stacking models comprising different combinations of the base learners were systematically evaluated. Following preliminary screening based on out-of-fold (OOF) performance within the training data, 16 candidate predictive models were retained for subsequent evaluation, including seven individual base classifiers, eight Stacking ensemble models, and one Soft Voting model.

**Figure 3:**
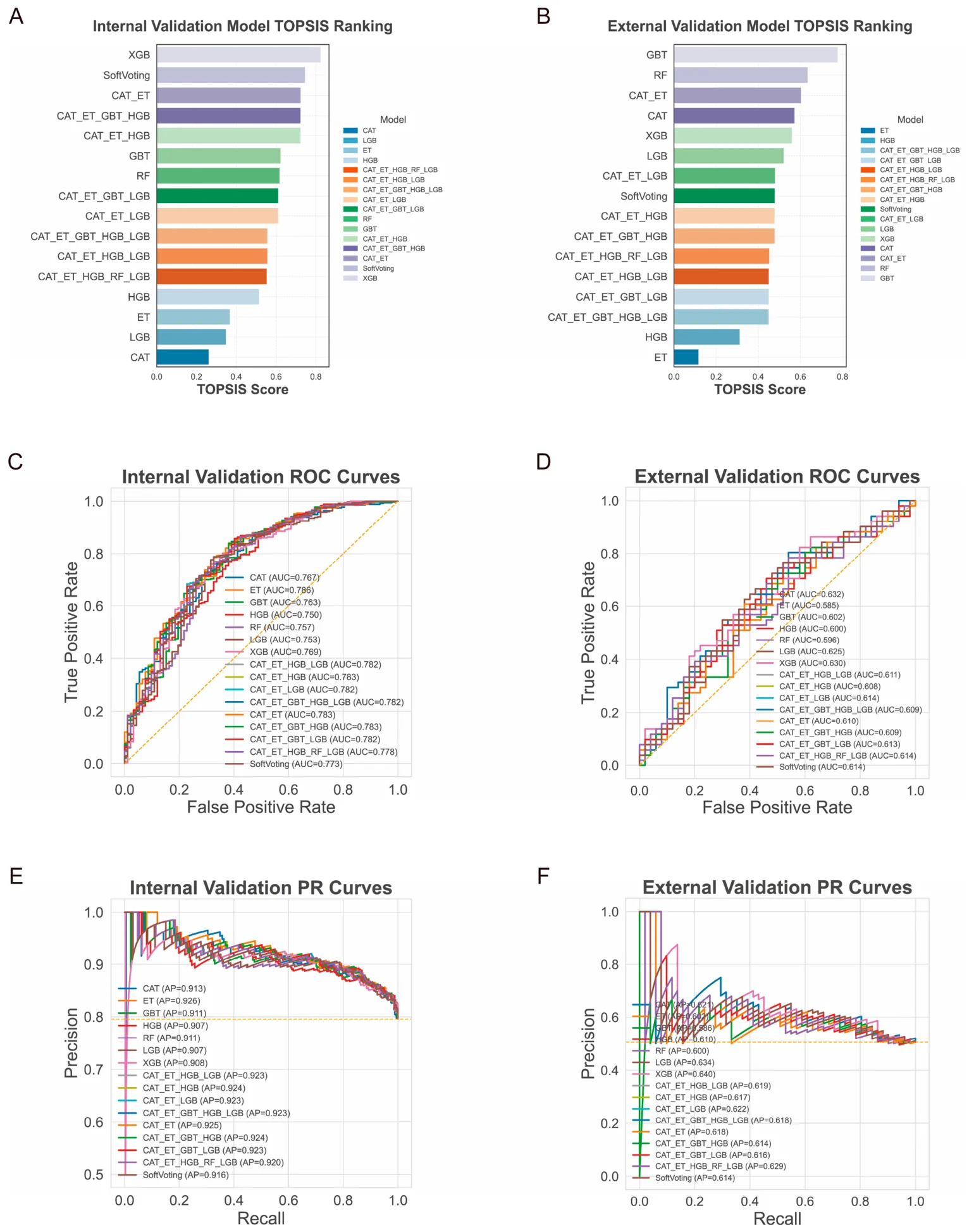
Performance evaluation and selection of candidate predictive models in the internal and independent external validation sets. (**A**) TOPSIS-based comprehensive ranking of the 16 candidate predictive models in the internal validation set. Eight performance metrics, including ROC-AUC, PR-AUC, accuracy, F1 score, positive predictive value (PPV), negative predictive value (NPV), sensitivity, and specificity, were incorporated into the TOPSIS analysis. (**B**) TOPSIS-based comprehensive ranking of the 16 candidate models in the independent external validation set using the same evaluation criteria. The three highest-ranked models in each evaluation dataset were retained, and the CAT+ET Stacking model, which was included among the Top 3 models in both datasets, was selected as the final predictive model according to the predefined intersection-based selection criterion. (**C**) Receiver operating characteristic (ROC) curves of the 16 candidate models in the internal validation set. (**D**) ROC curves of the candidate models in the independent external validation set. (**E**) Precision–recall (PR) curves of the candidate models in the internal validation set. (**F**) PR curves of the candidate models in the independent external validation set.

To avoid model selection based on a single performance measure, eight metrics—ROC-AUC, area under the precision–recall curve (PR-AUC), accuracy, F1 score, positive predictive value (PPV), negative predictive value (NPV), sensitivity, and specificity—were jointly incorporated into the Technique for Order Preference by Similarity to Ideal Solution (TOPSIS) analysis. The 16 candidate models were ranked separately according to their overall performance in the internal validation set and the independent external validation set (Figure 3A,B). ROC and PR curves were additionally generated to compare the discriminative performance of the candidate models across the two evaluation datasets (Figure 3C–F).

According to the predefined model-selection strategy, the three highest-ranked models based on the TOPSIS scores were retained separately from the internal and external validation sets, and the two Top-3 model sets were subsequently intersected. The CAT+ET Stacking model was ranked among the Top 3 models in both evaluation datasets and was therefore selected as the final predictive model according to this consensus-based selection criterion (Figure 3A,B). Notably, selection of the CAT+ET model was based on its integrated ranking across multiple performance metrics and its consistent inclusion among the highest-ranked candidates in both evaluation datasets, rather than on superiority in any single performance metric.

In the internal validation set, the CAT+ET model achieved a ROC-AUC of 0.783 (95% CI, 0.731–0.836) and a PR-AUC of 0.925 (95% CI, 0.896–0.950). The corresponding accuracy was 0.831 (95% CI, 0.796–0.864), F1 score was 0.901 (95% CI, 0.879–0.923), PPV was 0.844 (95% CI, 0.808–0.877), NPV was 0.700 (95% CI, 0.553–0.838), sensitivity was 0.966 (95% CI, 0.946–0.984), and specificity was 0.304 (95% CI, 0.211–0.400). The ROC and PR curves further illustrate the discriminative performance of the CAT+ET model relative to the other candidate models in the internal validation set (Figure 3C,E).

The cross-cohort generalizability of the model was subsequently evaluated in an external validation cohort that was completely excluded from pathway feature selection, model training, and hyperparameter optimization. In this independent cohort, the CAT+ET model yielded a ROC-AUC of 0.610 (95% CI, 0.496–0.720) and a PR-AUC of 0.618 (95% CI, 0.483–0.758). The corresponding accuracy was 0.525 (95% CI, 0.426–0.624), F1 score was 0.652 (95% CI, 0.550–0.738), PPV was 0.517 (95% CI, 0.409–0.619), NPV was 0.571 (95% CI, 0.294–0.846), sensitivity was 0.882 (95% CI, 0.789–0.964), and specificity was 0.160 (95% CI, 0.065–0.268). Compared with its performance in the internal validation set, the CAT+ET model showed reduced overall discrimination in the external cohort, particularly with respect to specificity. Notably, the 95% CI for the external ROC-AUC included 0.5, indicating limited cross-cohort generalizability and suggesting that inter-cohort heterogeneity remained an important challenge (Figure 3D,F).

To quantify uncertainty in the performance estimates, 95% confidence intervals for all performance metrics were calculated separately in the internal and independent external validation sets using 2000 nonparametric bootstrap resamples. Overall, the CAT+ET model demonstrated favorable discrimination and high sensitivity in the internal validation set, whereas independent external validation revealed reduced discrimination and specificity, indicating limited cross-cohort generalizability. Accordingly, CAT+ET was retained as the final predictive model on the basis of the predefined multi-metric consensus selection strategy and was subsequently subjected to SHAP analysis to characterize the contributions of the key pathway features to model predictions.

### 2.3. SHAP-Based Interpretation of the Final Predictive Model

**Figure 4:**
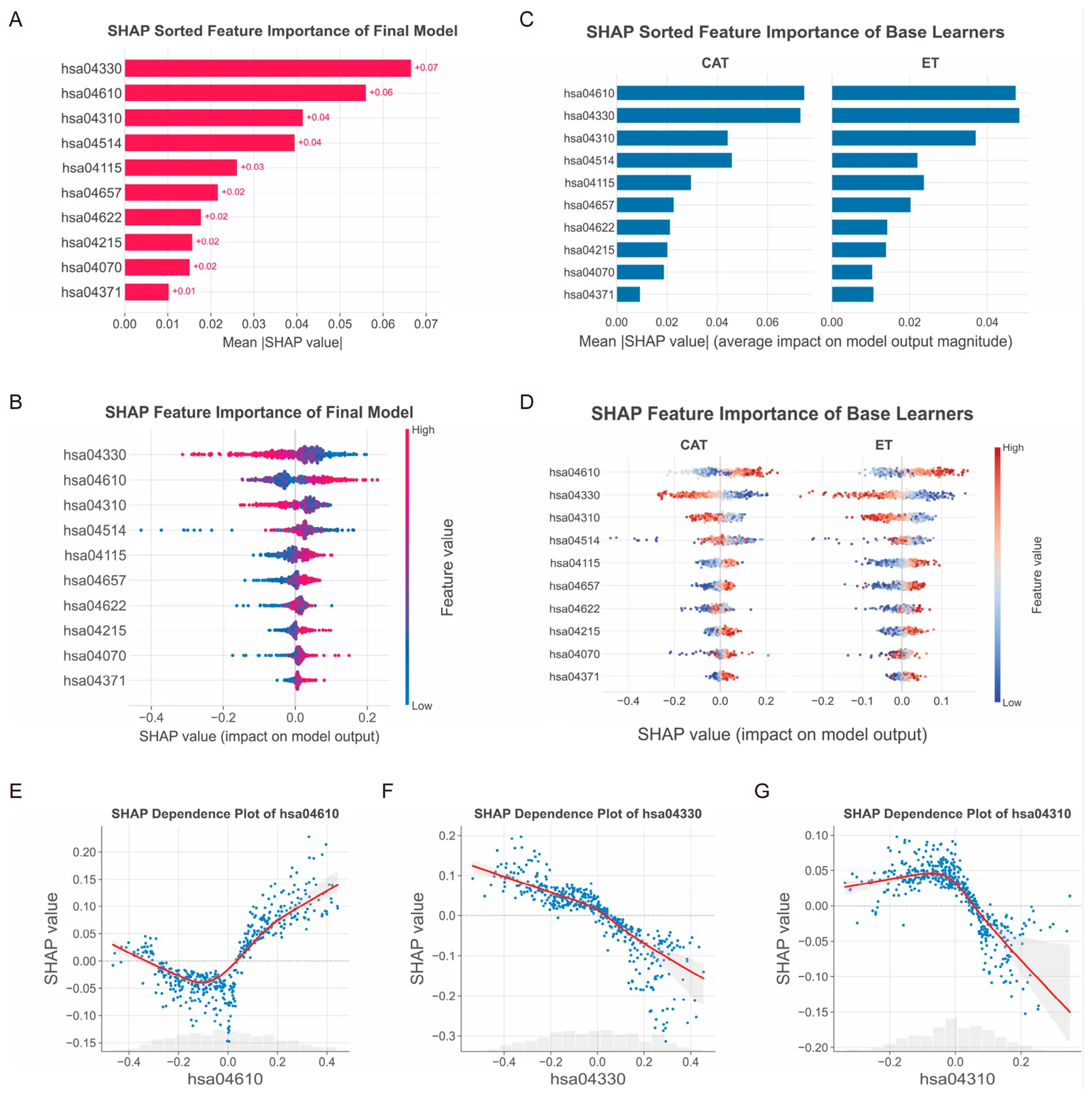
SHAP-based interpretation of the final CAT+ET ensemble predictive model. (**A**) Global importance ranking of the 10 key pathway features in the final CAT+ET model based on mean absolute SHAP values. (**B**) SHAP beeswarm plot showing the distribution of SHAP values for individual pathway features across samples. Each point represents an individual sample; the position along the *x*-axis indicates the magnitude and direction of the contribution of a pathway feature to model output, whereas the color represents the corresponding pathway feature value. (**C**) Global SHAP feature importance of the 10 pathway features in the CAT base learner. (**D**) Global SHAP feature importance of the 10 pathway features in the ET base learner. (**E**–**G**) SHAP dependence plots illustrating the relationships between pathway scores and SHAP values for Complement and coagulation cascades (hsa04610) (**E**), Notch signaling pathway (hsa04330) (**F**), and Wnt signaling pathway (hsa04310) (**G**). The dependence plots illustrate distinct pathway-activity-dependent patterns in their contributions to the final model output.

To characterize the predictive behavior of the final CAT+ET ensemble model and quantify the relative contributions of individual pathways to RA classification, we performed SHapley Additive exPlanations (SHAP) analysis. The global importance of the 10 key pathways was ranked according to their mean absolute SHAP values. The Notch signaling pathway (hsa04330) showed the highest mean absolute SHAP value (approximately 0.07), followed by Complement and coagulation cascades (hsa04610; approximately 0.06). The Wnt signaling pathway (hsa04310) and Cell adhesion molecules (hsa04514) also showed relatively high contributions, whereas the remaining pathways had lower mean absolute SHAP values (Figure 4A). Thus, hsa04330 and hsa04610 represented the two most influential pathway features in the final CAT+ET model.

SHAP beeswarm analysis was subsequently performed to characterize the relationships between pathway feature levels and model output (Figure 4B). The distributions of SHAP values varied substantially across individual samples, and different pathways exhibited distinct directional patterns between their feature levels and contributions to model predictions. These findings indicate that predictions generated by the final model were jointly determined by multiple pathway features rather than being dominated by a single pathway.

Because the final ensemble consisted of CAT and ET as its two constituent base learners, SHAP feature importance was further examined separately for each base learner to assess the consistency of pathway contributions across model architectures (Figure 4C,D). Complement and coagulation cascades (hsa04610) and the Notch signaling pathway (hsa04330) ranked among the most influential features in both CAT and ET. In CAT, the mean absolute SHAP value of hsa04610 was slightly higher than that of hsa04330, whereas both pathways showed high feature importance in ET. The Wnt signaling pathway (hsa04310) also exhibited a relatively high contribution in both base learners. These findings indicate that the prominent contributions of hsa04330 and hsa04610 to the final ensemble were not driven exclusively by a single base learner but were consistently observed across both CAT and ET.

To further characterize the relationship between pathway activity and model output, SHAP dependence analyses were performed for the pathways with the highest global SHAP importance (Figure 4E–G). Complement and coagulation cascades (hsa04610) exhibited a pronounced nonlinear relationship between pathway score and SHAP value. As the hsa04610 pathway score increased from relatively low levels, its SHAP value initially decreased; with further increases in pathway score, however, the SHAP value increased markedly and gradually shifted from negative to positive values at higher pathway scores (Figure 4E). In contrast, the Notch signaling pathway (hsa04330) showed an overall decreasing relationship between pathway score and SHAP value (Figure 4F). The Wnt signaling pathway (hsa04310) also displayed a nonlinear pattern, with relatively limited changes in SHAP values at lower pathway scores followed by a marked decrease once the pathway score increased beyond a certain range (Figure 4G). Collectively, these findings indicate that the contributions of key pathways to model output followed distinct activity-dependent patterns.

Considering both global SHAP importance and SHAP dependence patterns, hsa04610 not only showed a high global contribution to the final CAT+ET model but also exhibited a distinct nonlinear and pathway-activity-dependent association with model output. In particular, at higher pathway scores, increasing hsa04610 activity was accompanied by a transition from negative to positive SHAP values and progressively greater positive contributions to RA prediction. Based on its high global SHAP importance together with this characteristic nonlinear predictive pattern, hsa04610 was therefore carried forward as a candidate pathway for subsequent target-prioritization and biological analyses.

### 2.4. Identification of Potential Therapeutic Targets Within the Key Pathway

**Figure 5:**
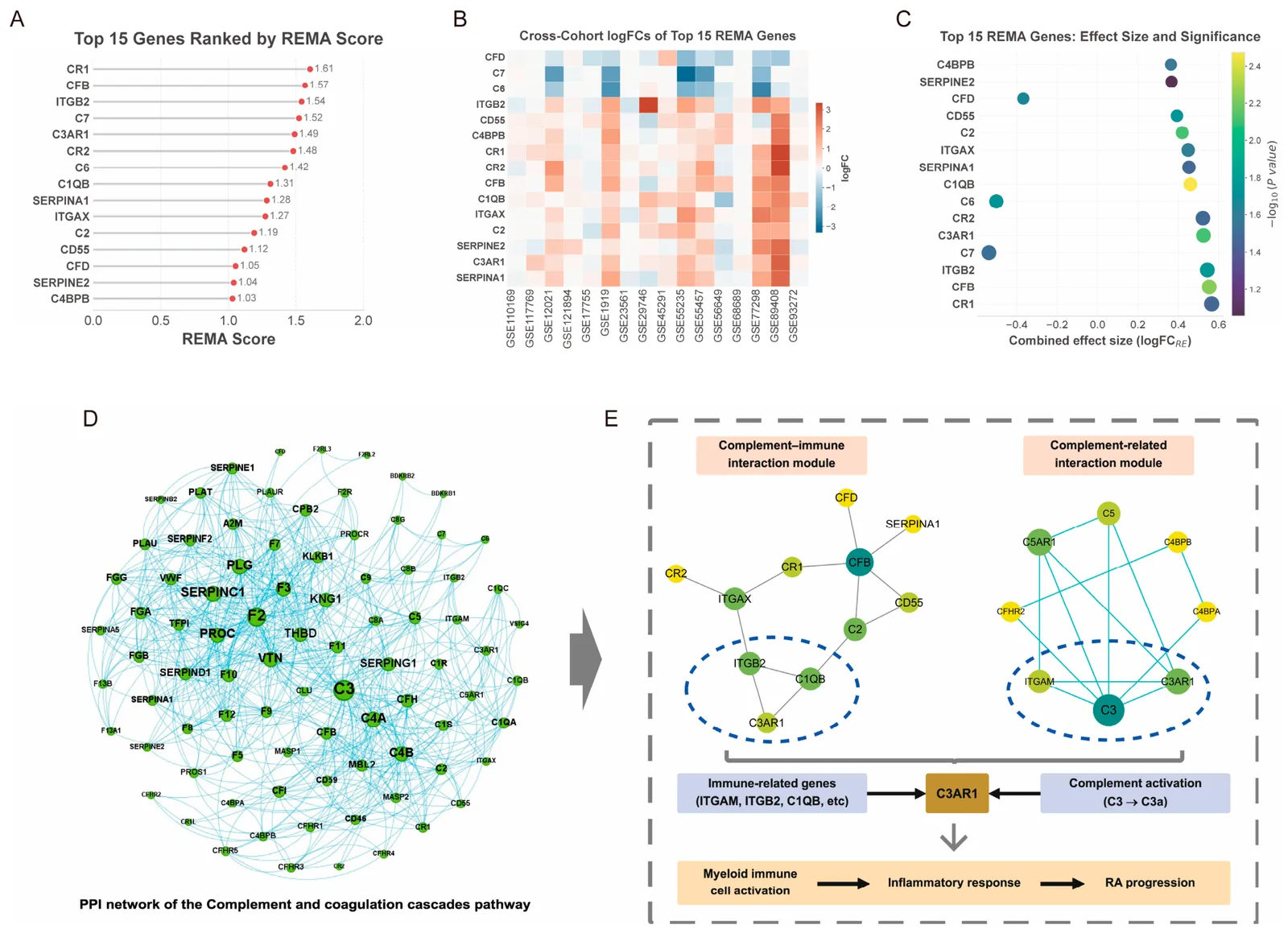
Integrated prioritization of candidate genes and identification of C3AR1 within the Complement and coagulation cascades pathway. (**A**) Top 15 genes within the Complement and coagulation cascades pathway (hsa04610) ranked according to their REMA scores. (**B**) Heatmap showing the cross-cohort log fold-change (logFC) profiles of the Top 15 REMA-ranked genes across the included GEO datasets. (**C**) Combined effect sizes estimated by the random-effects model and their corresponding statistical significance for the Top 15 REMA-ranked genes. (**D**) PPI network of genes associated with the Complement and coagulation cascades pathway. (**E**) Integrated network and biological-context analysis of the Top 15 REMA-ranked genes. Densely interconnected modules were identified from the PPI network using the MCODE plugin in Cytoscape, and network relationships, MCODE-derived module composition, and complement–immune inflammatory context were considered together for candidate-target prioritization.

Based on the pathway-level interpretability analysis described above, the Complement and coagulation cascades pathway (hsa04610) was carried forward for target-level investigation. Genes within hsa04610 were subjected to random-effects meta-analysis (REMA) to integrate gene-expression alterations across independent GEO cohorts. Candidate genes were ranked according to the REMA score, which jointly reflected cross-cohort effect estimates and statistical evidence. CR1, CFB, ITGB2, C7, and C3AR1 were among the highest-ranked genes, with REMA scores of 1.61, 1.57, 1.54, 1.52, and 1.49, respectively, followed by CR2, C6, C1QB, SERPINA1, ITGAX, and other candidates (Figure 5A). Thus, C3AR1 represented one of the highly ranked candidates within hsa04610 rather than the top-ranked gene according to the REMA score.

To further characterize the cross-cohort consistency of these candidate genes, the logFC profiles of the Top 15 REMA-ranked genes were compared across the GEO datasets (Figure 5B). Although a degree of inter-cohort heterogeneity was observed, recurrent expression alterations were evident for multiple complement- and immune-related genes across independent datasets. We further examined the combined effect sizes and statistical significance derived from the random-effects models (Figure 5C). Several highly ranked genes, including CR1, CFB, ITGB2, C7, C3AR1, and CR2, showed positive pooled effect estimates together with statistical support, providing additional cross-cohort evidence for their prioritization within hsa04610.

Because REMA primarily captures integrated expression evidence across independent cohorts and does not by itself define the functional positions of individual genes within the pathway network, the expression-based evidence was further integrated with protein–protein interaction information. A protein–protein interaction (PPI) network was constructed for genes associated with the Complement and coagulation cascades pathway using the STRING database (version 12.0), with a combined score > 0.7 used as the interaction confidence threshold. The resulting network revealed extensive interactions among complement components, complement receptors, and immune-related molecules (Figure 5D). The Top 15 REMA-ranked genes were subsequently mapped onto the PPI network, and densely interconnected network modules were identified using the MCODE plugin (version 2.0.3) in Cytoscape (version 3.7.2). The interaction relationships among the Top 15 REMA-ranked genes and the composition of the MCODE-identified modules were then considered together with their complement–immune inflammatory context for subsequent candidate-target prioritization (Figure 5E).

This integrated analysis revealed interconnected molecular contexts involving complement activation and immune-related processes. C3AR1 was positioned within this integrated network at an interface linking complement-associated signaling with immune-related molecular interactions. In particular, the network context connected C3AR1 with C3-associated complement activation while also placing it in relation to immune-associated molecules represented within the REMA-enriched interaction modules (Figure 5E). These findings provided network- and mechanism-level information complementary to the cross-cohort expression evidence obtained from REMA.

Accordingly, C3AR1 was not prioritized solely on the basis of its REMA ranking or any single network-topological measure. Although its REMA score (1.49) was lower than those of CR1, CFB, ITGB2, and C7, C3AR1 was supported by convergent evidence from cross-cohort expression analysis, REMA-based prioritization, PPI network relationships, and MCODE-derived module analysis. Moreover, previous studies have implicated C3AR1 in complement-mediated immune and inflammatory processes [25,26,27], providing independent biological support for the network- and expression-based evidence obtained in the present study. Considering these complementary lines of evidence, C3AR1 was carried forward as the candidate target for subsequent structure-based virtual screening and experimental investigation.

### 2.5. Molecular Docking Screening of Candidate Natural Small Molecules

A molecular virtual library containing approximately 470,000 natural-product small molecules was constructed. Subsequently, structure-based virtual screening was performed against C3AR1 (C3a anaphylatoxin chemotactic receptor; UniProt ID: Q16581) as the candidate receptor. The three-dimensional structure of C3AR1 was obtained from the Protein Data Bank (PDB ID: 8ZWF). The 8ZWF structure represents a JR14A-bound, BRIL-engineered active-state conformation of C3aR and provides an experimentally resolved ligand-bound receptor conformation with a structurally defined ligand-accessible region; it was therefore used for subsequent structure-based virtual screening and generation of candidate binding poses. However, self-redocking of JR14A yielded a heavy-atom RMSD of 3.88 Å for the top-ranked pose relative to the crystallographic pose, indicating limited recovery of the experimentally observed binding orientation. A closer pose (RMSD = 2.18 Å) was sampled but was not ranked first, suggesting limitations in pose ranking by the docking scoring function.

**Figure 6:**
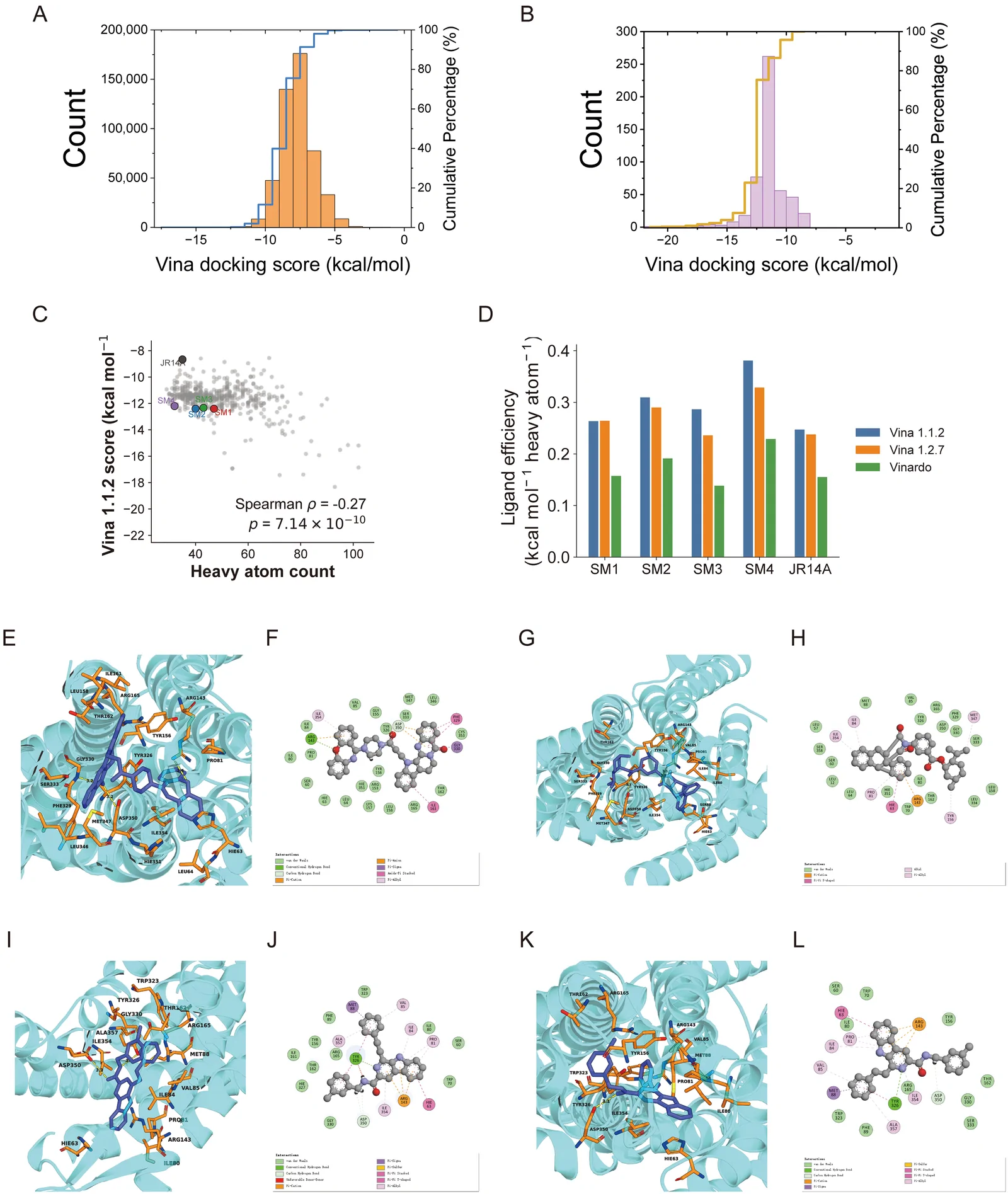
Virtual screening and molecular docking analysis of candidate natural compounds against C3AR1. (**A**) Distribution and cumulative distribution of docking scores obtained from the preliminary large-scale virtual screening using Vina-GPU 2.1. (**B**) Distribution and cumulative distribution of mean Vina 1.1.2 docking scores for the top 500 compounds subjected to refined docking. (**C**) Relationship between ligand heavy-atom count and mean Vina 1.1.2 docking score among the 500 compounds subjected to refined docking. SM1–SM4 and the structure-derived reference ligand JR14A are highlighted. Spearman’s correlation coefficient (ρ) and corresponding *p* value are shown. (**D**) Ligand efficiency (LE) of SM1–SM4 and JR14A calculated from the mean docking scores obtained using Vina 1.1.2, Vina 1.2.7, and Vinardo. (**E**,**F**) Three-dimensional predicted binding pose and two-dimensional interaction diagram of SM1 with C3AR1, respectively. (**G**,**H**) Corresponding three-dimensional and two-dimensional analyses of SM2. (**I**,**J**) Corresponding analyses of SM3. (**K**,**L**) Corresponding analyses of SM4.

First, large-scale preliminary virtual screening of the natural-product library was performed using Vina-GPU 2.1, and the candidate compounds were ranked according to their docking scores. As shown in Figure 6A, the docking scores of most screened molecules were predominantly distributed between −8 and −6 kcal/mol, with the cumulative distribution indicating that approximately 70–80% of the compounds fell within this range.

Based on the preliminary screening results, the top 500 candidate molecules were selected for refined molecular docking using AutoDock Vina 1.1.2. As shown in Figure 6B, the mean docking scores of the Top-500 candidates were predominantly distributed between −12 and −10 kcal/mol, with several compounds exhibiting scores approaching −20 kcal/mol. The cumulative distribution further showed that approximately 80–90% of these candidates had docking scores below −10 kcal/mol, indicating an overall enrichment toward more negative Vina 1.1.2 scores among the compounds entering the refined-docking stage.

Given the exceptionally negative docking scores observed for some compounds, the potential influence of molecular size on the raw Vina 1.1.2 docking scores was further examined. Across the 500 compounds subjected to refined docking, ligand heavy-atom count showed a weak negative correlation with the mean Vina 1.1.2 docking score (Spearman ρ = −0.271, *p* = 7.14 × 10^−10^; Figure 6C). Within the Vina 1.1.2 Top-100 subset, the association was more pronounced (ρ = −0.484, *p* = 3.32 × 10^−7^). LE was subsequently evaluated as a size-normalized complementary metric. LE values calculated from the scores obtained using Vina 1.1.2, Vina 1.2.7, and Vinardo showed that the apparent raw-score advantage of some larger compounds was reduced after normalization by heavy-atom count (Figure 6D).

To further evaluate the consistency of compound rankings across different scoring approaches, the Top-100 compounds selected according to Vina 1.1.2 were additionally assessed using Vina 1.2.7 and Vinardo, and a rank-based consensus score was calculated from the three individual rankings. Relative compound rankings varied to some extent across the scoring approaches. Among the four compounds ultimately advanced for downstream analyses, SM1 showed the greatest cross-method ranking consistency, ranking 62nd, 64th, and 69th with Vina 1.1.2, Vina 1.2.7, and Vinardo, respectively, yielding a consensus rank score of 65.00 and an overall consensus rank of 64. The corresponding consensus rank scores for SM2, SM3, and SM4 were 72.33, 90.67, and 84.67, with overall consensus ranks of 78, 98, and 93, respectively. JR14A, the co-crystallized ligand in the 8ZWF structure, was included as a structure-derived reference ligand in the docking-score and LE analyses but was not included in the consensus ranking of the Top-100 natural-product candidates.

Based on the refined-docking results, the Top-100 compounds ranked by the Vina 1.1.2 docking score were retained as the initial candidate pool for further evaluation. Their predicted binding poses were subsequently subjected to qualitative visual inspection to assess plausible accommodation within the predefined C3AR1 ligand-binding region and reasonable ligand–receptor interaction geometries. This step served primarily to exclude obviously implausible predicted poses rather than as an additional quantitative reranking procedure. Practical compound availability was subsequently considered. Because some of the identified natural products were not commercially available or could not be practically obtained, four obtainable compounds (SM1–SM4) were ultimately selected for subsequent computational characterization and in vitro evaluation. The basic information and docking scores obtained using Vina 1.1.2, Vina 1.2.7, and Vinardo for the four candidate compounds are summarized in Table 2.

Three-dimensional binding-pose analysis showed that SM1–SM4 adopted spatially plausible predicted poses within the predefined C3AR1 ligand-binding region (Figure 6E,G,I,K). Although the predicted orientations varied among the candidate compounds, each compound formed multiple non-covalent contacts with receptor residues within this region. Two-dimensional interaction analysis (Figure 6F,H,J,L) further revealed predicted hydrogen bonds, hydrophobic contacts, and π-related interactions between the candidate compounds and C3AR1. Several residues, including ARG143, TYR156, and ARG165, recurred in the predicted interactions across multiple candidate complexes.

Collectively, the virtual-screening results, ligand-size analysis, ligand-efficiency evaluation, cross-method scoring assessment, and predicted binding-pose analysis supported the advancement of SM1–SM4 to subsequent molecular dynamics simulations and experimental evaluation.

To further characterize the developability of the four candidate compounds, their physicochemical properties, drug-likeness, and absorption, distribution, metabolism, excretion, and toxicity (ADMET) profiles were evaluated using SwissADME and ADMETlab 3.0 (Supplementary Data S8). SwissADME predicted high gastrointestinal absorption and no Veber violations for all four compounds, whereas SM1 and SM2 showed two Lipinski violations and SM3 and SM4 showed one violation each. ADMETlab 3.0 provided complementary predictions of ADMET-related properties. Overall, these in silico assessments revealed several potentially favorable properties together with compound-specific limitations, providing additional information for the subsequent evaluation of SM1–SM4.

### 2.6. Molecular Dynamics Simulation Analysis of Candidate Ligand–C3AR1 Complexes

**Figure 7:**
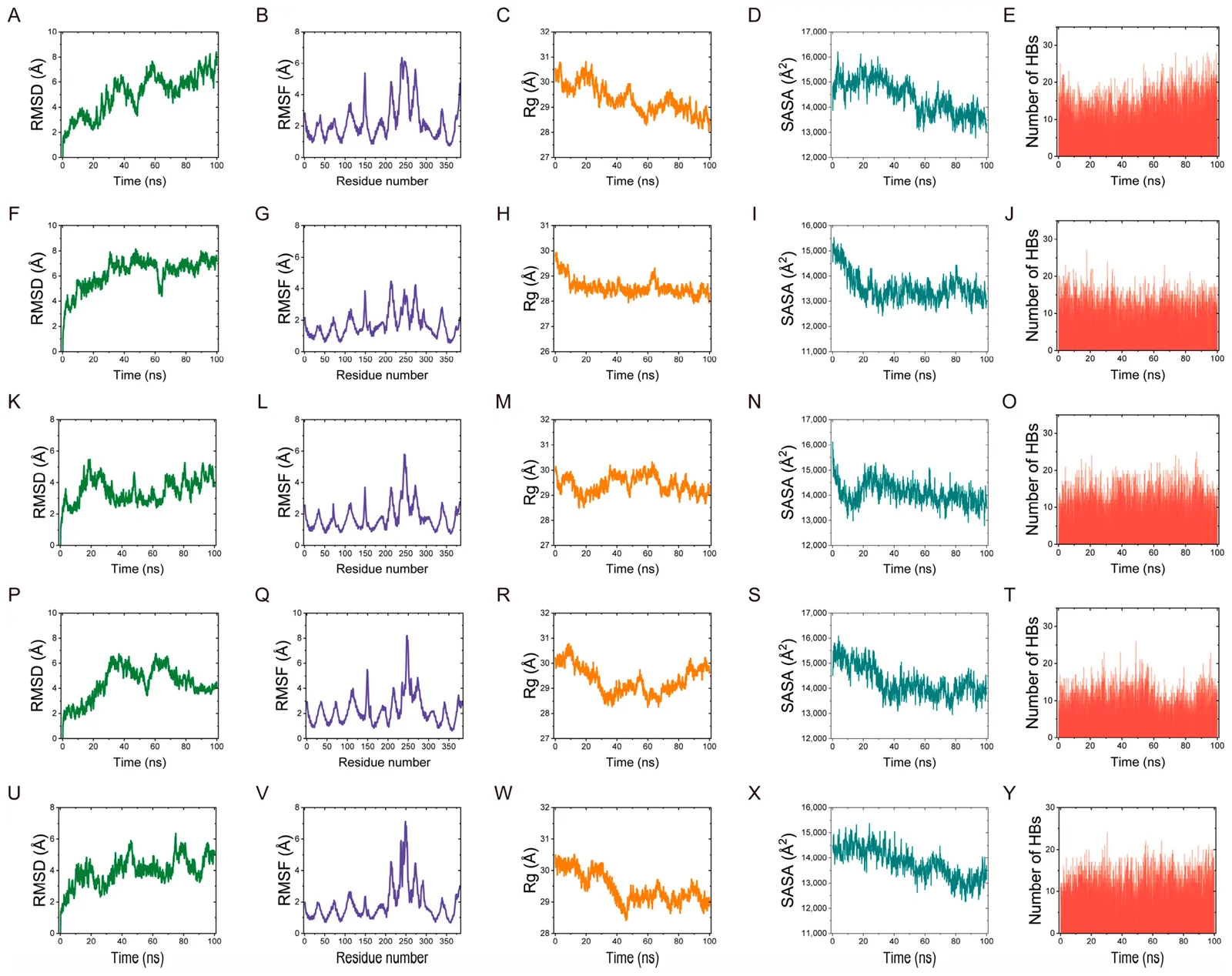
Structural dynamics of C3AR1–ligand complexes during 100-ns molecular dynamics simulations in an explicit lipid bilayer environment. The dynamic behavior of C3AR1 in complex with four candidate natural small molecules (SM1–SM4) and the reference ligand JR14A was evaluated throughout 100-ns production MD simulations. (**A**–**E**) SM1–C3AR1; (**F**–**J**) SM2–C3AR1; (**K**–**O**) SM3–C3AR1; (**P**–**T**) SM4–C3AR1; and (**U**–**Y**) JR14A–C3AR1. Within each ligand-bound system, the panels show the root mean square deviation (RMSD) of the complex, root mean square fluctuation (RMSF) of receptor residues, radius of gyration (Rg), solvent-accessible surface area (SASA), and number of intermolecular hydrogen bonds (HBs), respectively. All simulations were performed using membrane-embedded C3AR1 systems to account for the native transmembrane environment of the receptor.

To further characterize the dynamic behavior and interaction energetics of the selected ligand–receptor complexes under a membrane-associated environment, 100-ns explicit-lipid-bilayer MD simulations were performed for the four candidate compounds (SM1–SM4), with the experimentally resolved ligand JR14A included as a reference system. Structural dynamics were first evaluated using protein Cα root mean square deviation (RMSD), root mean square fluctuation (RMSF), Rg, solvent-accessible surface area (SASA), and intermolecular hydrogen-bond analyses (Figure 7). The protein Cα RMSD trajectories revealed distinct conformational adjustment patterns among the five systems (Figure 7A,F,K,P,U). Over the complete trajectories, the mean RMSD values were 5.02 ± 1.60, 6.23 ± 1.19, 3.49 ± 0.80, 4.39 ± 1.32, and 3.96 ± 0.91 Å for the SM1–C3AR1, SM2–C3AR1, SM3–C3AR1, SM4–C3AR1, and JR14A–C3AR1 systems, respectively. During the final 20 ns, SM3 and SM4 exhibited mean RMSD values of 4.19 ± 0.52 and 4.09 ± 0.36 Å, respectively, which were comparable in magnitude to that of the JR14A reference system (4.52 ± 0.65 Å). In contrast, SM1 and SM2 maintained higher RMSD levels during the same period, with mean values of 6.66 ± 0.70 and 7.02 ± 0.42 Å, respectively. Notably, SM4 underwent a relatively pronounced conformational adjustment during the earlier stages of the trajectory, followed by a reduction in RMSD during the later stage. These observations indicate ligand-dependent differences in the extent of receptor conformational rearrangement rather than uniform dynamic behavior across the five systems.

Residue-level RMSF analysis further demonstrated that structural fluctuations were nonuniformly distributed along the receptor sequence (Figure 7B,G,L,Q,V). The mean RMSF values were 2.29, 1.72, 1.78, 2.26, and 1.87 Å for the SM1-, SM2-, SM3-, SM4-, and JR14A-bound systems, respectively. Larger fluctuations were predominantly localized to flexible receptor regions, whereas substantial portions of the receptor exhibited comparatively lower residue-level mobility. SM2 and SM3 showed relatively lower overall residue fluctuations, whereas SM1 and SM4 exhibited greater local flexibility. Thus, the differences observed in global RMSD were accompanied by system-dependent local conformational dynamics rather than uniform structural destabilization of the receptor.

The Rg profiles remained within relatively restricted ranges throughout the simulations (Figure 7C,H,M,R,W), with mean values of 29.32 ± 0.57, 28.55 ± 0.32, 29.42 ± 0.35, 29.33 ± 0.55, and 29.44 ± 0.48 Å for the SM1-, SM2-, SM3-, SM4-, and JR14A-bound systems, respectively. During the final 20 ns, the corresponding Rg values were 28.74 ± 0.28, 28.34 ± 0.17, 29.17 ± 0.22, 29.64 ± 0.30, and 29.17 ± 0.22 Å, respectively. The SASA profiles similarly revealed system-dependent changes in receptor solvent exposure (Figure 7D,I,N,S,X). Over the complete trajectories, the mean SASA values were 14,406.95 ± 684.01, 13,527.68 ± 547.02, 14,075.90 ± 436.71, 14,273.01 ± 604.66, and 13,791.01 ± 604.34 Å^2^ for the SM1-, SM2-, SM3-, SM4-, and JR14A-bound systems, respectively. During the final 20 ns, the corresponding values were 13,649.35 ± 291.50, 13,380.34 ± 354.82, 13,779.26 ± 330.08, 14,004.46 ± 319.50, and 13,069.60 ± 341.65 Å^2^, respectively. Compared with their full-trajectory averages, all five systems showed lower mean SASA values during the final 20 ns, although the magnitude of this change differed among systems. Together with the relatively restricted Rg profiles, these results indicate that the receptor maintained an overall compact architecture while undergoing ligand-dependent adjustments in compactness and solvent exposure during the simulations.

Intermolecular hydrogen bonds were dynamically formed and disrupted throughout the trajectories (Figure 7E,J,O,T,Y), indicating continuous reorganization of polar receptor–ligand contacts. The mean numbers of hydrogen bonds were 14.93 ± 3.99, 12.81 ± 3.24, 13.15 ± 3.42, 11.16 ± 3.28, and 12.85 ± 3.06 for the SM1-, SM2-, SM3-, SM4-, and JR14A-bound systems, respectively. Among the candidate systems, SM1 displayed the highest mean hydrogen-bond count and reached an average of 17.96 ± 3.76 during the final 20 ns. Nevertheless, because hydrogen-bond number alone does not define overall binding stability, these observations were interpreted together with the structural and energetic analyses.

**Figure 8:**
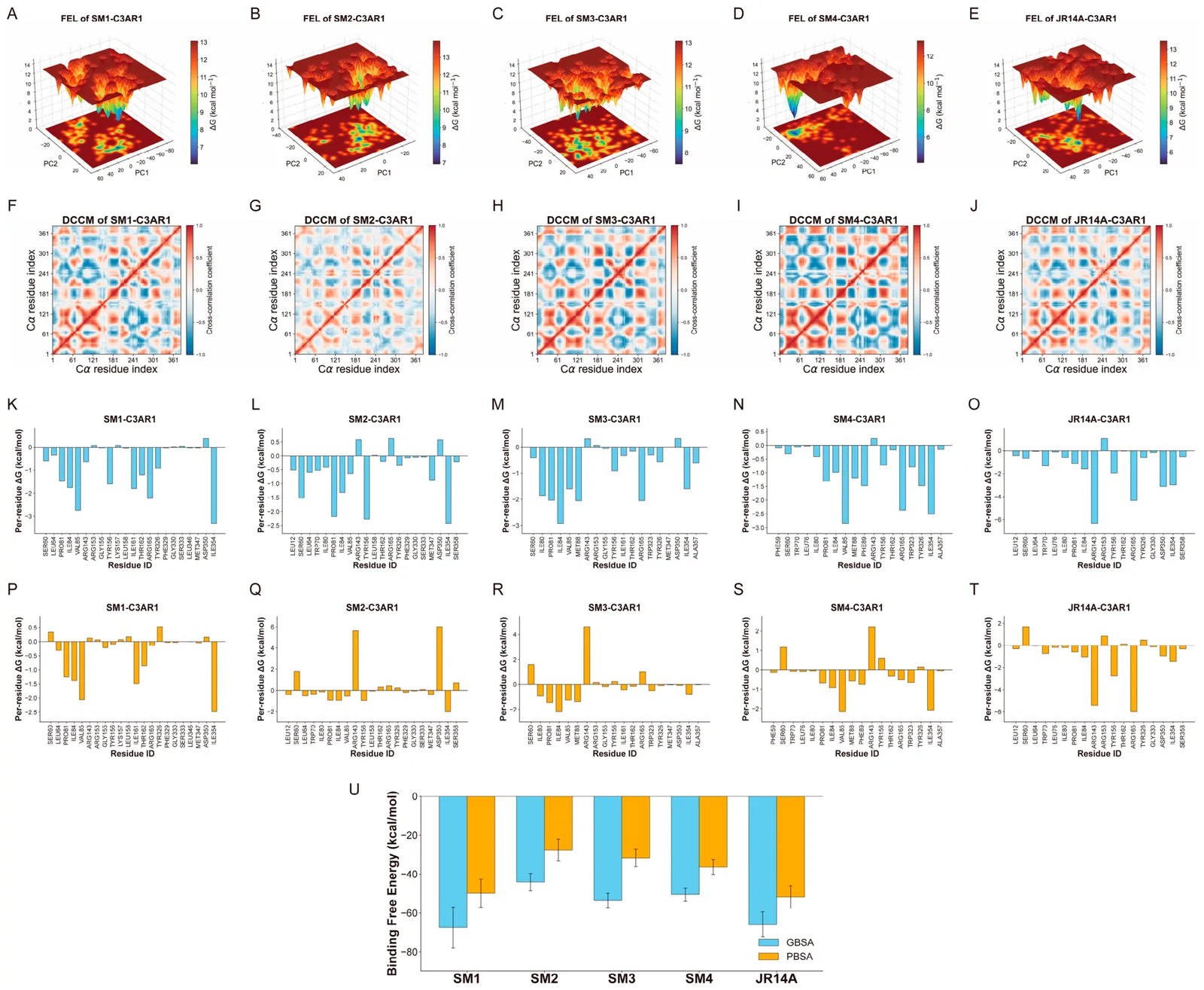
Conformational dynamics and binding energetics of C3AR1–ligand complexes derived from explicit-membrane molecular dynamics simulations. (**A**–**E**) Free-energy landscapes (FELs) projected onto the first two principal components (PC1 and PC2) for the SM1–C3AR1, SM2–C3AR1, SM3–C3AR1, SM4–C3AR1, and JR14A–C3AR1 systems, respectively. (**F**–**J**) Dynamic cross-correlation matrices (DCCMs) illustrating correlated and anticorrelated motions of C3AR1 residues in the corresponding five ligand-bound systems. (**K**–**O**) Per-residue binding free-energy decomposition calculated using the MM/GBSA approach for SM1, SM2, SM3, SM4, and JR14A, respectively. (**P**–**T**) Corresponding per-residue energy decomposition calculated using the MM/PBSA approach. (**U**) Comparison of the overall MM/GBSA- and MM/PBSA-derived binding free energies of SM1–SM4 and JR14A with C3AR1. Negative values indicate energetically favorable calculated receptor–ligand association. JR14A was included as a reference ligand to facilitate comparative evaluation of the candidate compounds.

To further characterize conformational sampling beyond conventional trajectory descriptors, principal component analysis (PCA)-based Gibbs free-energy landscapes were constructed using the first two principal components (PC1 and PC2) (Figure 8A–E). The five systems exhibited distinct distributions of low-free-energy conformational states, with differences in the location and population of the sampled energy basins. These patterns indicate that association with different ligands was accompanied by distinct conformational sampling of C3AR1 during the simulations. Consistent with these observations, dynamic cross-correlation matrix (DCCM) analysis revealed system-dependent patterns of correlated and anticorrelated residue motions (Figure 8F–J), indicating differences in the organization of collective receptor dynamics among the ligand-bound systems. Together, the free-energy landscape (FEL) and DCCM analyses complemented the RMSD and RMSF results by demonstrating differences not only in the magnitude of structural fluctuations but also in the conformational states and collective motions sampled by the receptor.

Binding energetics were subsequently evaluated using both molecular mechanics/generalized Born surface area (MM/GBSA) and molecular mechanics/Poisson–Boltzmann surface area (MM/PBSA) calculations (Figure 8U). Negative binding-energy estimates were obtained for all four candidate complexes and the JR14A reference system. Among the candidate compounds, MM/GBSA energies ranged from −44.11 ± 4.40 to −67.55 ± 10.50 kcal/mol, whereas MM/PBSA energies ranged from −27.68 ± 5.70 to −49.90 ± 7.20 kcal/mol. SM1 showed the most favorable calculated binding energetics among the four candidate compounds, with MM/GBSA and MM/PBSA values of −67.55 ± 10.50 and −49.90 ± 7.20 kcal/mol, respectively. These values were broadly comparable in magnitude to those obtained for the JR14A reference system (−65.80 ± 6.38 and −51.84 ± 5.81 kcal/mol, respectively). SM2, SM3, and SM4 yielded MM/GBSA values of −44.11 ± 4.40, −53.59 ± 3.68, and −50.56 ± 3.41 kcal/mol, respectively, with corresponding MM/PBSA values of −27.68 ± 5.70, −31.70 ± 4.44, and −36.41 ± 3.75 kcal/mol, respectively. Notably, SM1 and JR14A showed similar energetic magnitudes across both approaches, although their relative ordering differed between MM/GBSA and MM/PBSA. Accordingly, these calculated energies were interpreted as comparative energetic estimates within the present simulation framework rather than as direct measures of experimental binding affinity.

Per-residue free-energy decomposition using both MM/GBSA and MM/PBSA further revealed recurrent energetic contributions from residues within the ligand-interaction region (Figure 8K–T). Residues including Ser60, Pro81, Ile84, Arg143, Tyr156, Thr162, Arg165, Tyr326, and Ile354 recurrently appeared among the contributing residues across the five systems, while additional energetic contributions varied among individual ligands. This pattern indicates that the candidate compounds and JR14A shared portions of the receptor interaction environment while retaining ligand-dependent energetic fingerprints. The residue-level results therefore provide complementary information to the total binding-energy estimates rather than indicating an identical interaction pattern across all five systems.

Taken together, the membrane MD simulations revealed distinct but sustained dynamic interaction patterns among the four candidate C3AR1 complexes. SM3 and SM4 exhibited relatively lower protein Cα RMSD values during the later stage of the simulations, whereas SM1 showed the most favorable calculated binding energetics among the candidate compounds and the highest mean number of intermolecular hydrogen bonds. Notably, the MM/GBSA and MM/PBSA estimates for SM1 were broadly comparable in magnitude to those of the structure-derived JR14A reference system. The FEL, DCCM, and per-residue energy-decomposition analyses further demonstrated both shared and ligand-dependent features of receptor–ligand interactions. Collectively, these results provide complementary structural, dynamic, and energetic evidence supporting the compatibility of the screened compounds with the C3AR1 binding environment, while not by themselves establishing receptor inhibition or experimental target engagement.

### 2.7. In Vitro Evaluation of Candidate Natural Small Molecules

**Figure 9:**
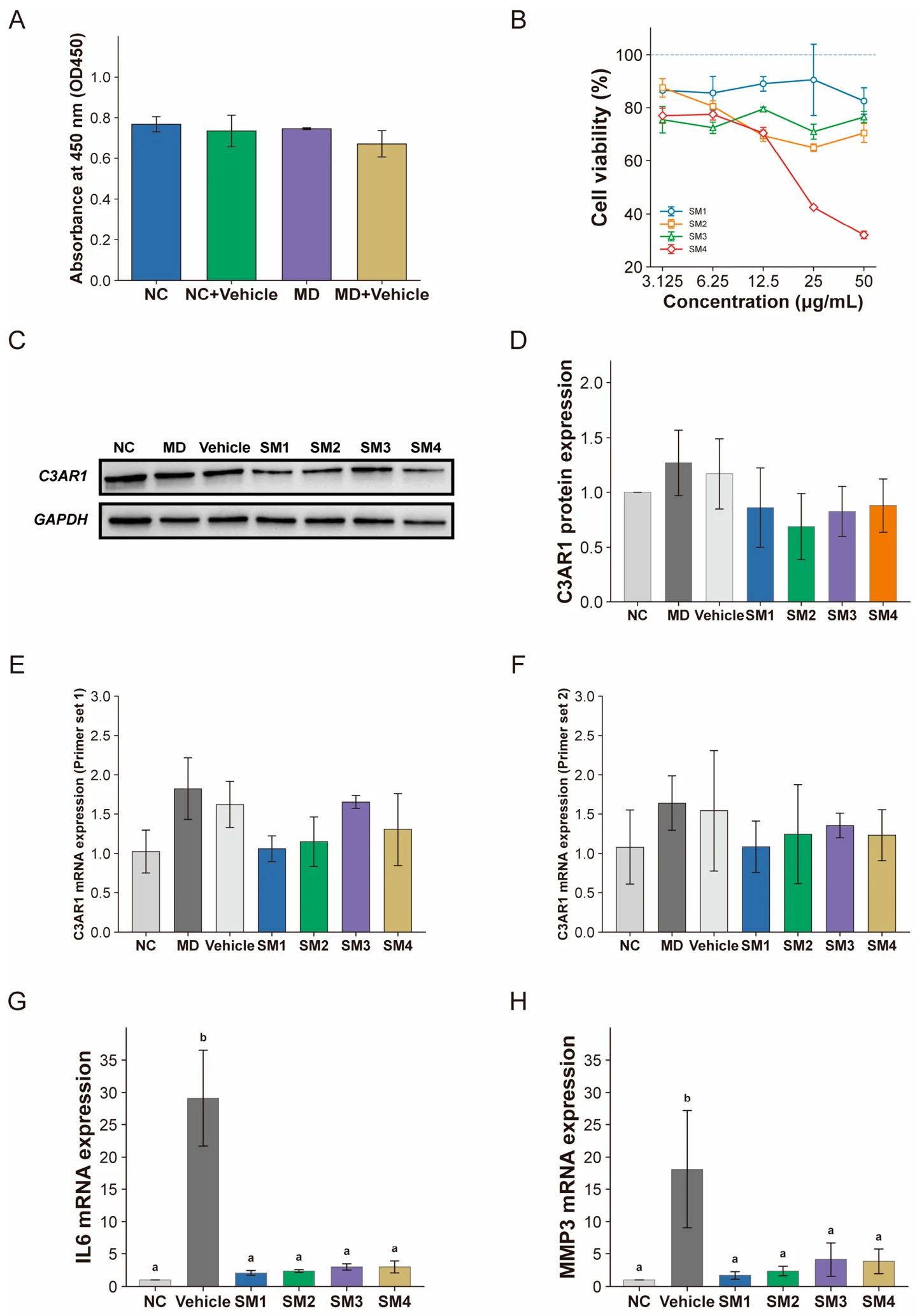
In vitro evaluation of four candidate natural small molecules for their effects on C3AR1 expression and inflammatory responses. (**A**) Cell viability analysis of different experimental groups using the CCK-8 assay. (**B**) Dose-dependent effects of the candidate natural small molecules on cell viability after treatment at different concentrations (3.125, 6.25, 12.5, 25, and 50 μg/mL). (**C**,**D**) Western blot analysis and quantitative evaluation of C3AR1 protein expression following treatment with the candidate natural small molecules. Compound-dependent numerical changes in C3AR1 protein expression were observed across the treatment groups. Quantitative data in (**D**) are presented as mean ± SD from four independent biological experiments (*n* = 4), with an overall one-way ANOVA *p* value of 0.0816. (**E**,**F**) Relative *C3AR1* mRNA expression in different treatment groups determined by RT-qPCR using two independent primer pairs. Candidate-compound treatment was associated with varying numerical changes in *C3AR1* mRNA expression across the experimental groups. Data are presented as mean ± SD from three independent biological experiments (*n* = 3). For primer pair 1 (**E**), one-way ANOVA indicated a significant overall difference among the groups (*p* = 0.0306), although individual pairwise comparisons did not reach statistical significance after Tukey’s multiple-comparisons correction (all adjusted *p* > 0.05). For primer pair 2 (**F**), the overall group difference was not statistically significant (*p* > 0.05). (**G**,**H**) Relative mRNA expression levels of the inflammation-related genes interleukin-6 (IL6) and matrix metalloproteinase 3 (MMP3) following treatment with the candidate natural small molecules. Candidate-compound treatment produced differential changes in *IL6* and *MMP3* expression in TNF-α-stimulated MH7A cells. Different lowercase letters indicate statistically significant differences between groups (*p* < 0.05), whereas groups sharing the same letter are not significantly different. The dashed line represents the control baseline (100% cell viability).

The four candidate natural small molecules (SM1–SM4) were further evaluated in a TNF-α-induced MH7A inflammatory model. Cell viability was first assessed using the Cell Counting Kit-8 (CCK-8) assay to determine suitable concentrations for subsequent experiments (Figure 9A,B). Considering both the cell viability results and the requirements of subsequent functional assays, SM1, SM3, and SM4 (12.5 μg/mL) and SM2 (6.25 μg/mL) were selected as the working concentrations for subsequent experiments.

As shown in Figure 9C,D, Western blot analysis was performed to evaluate changes in C3AR1 protein expression following treatment with the candidate natural small molecules. Compared with the normal control (NC) group, the model (MD) group stimulated with TNF-α showed a numerical increase in C3AR1 protein expression. Meanwhile, C3AR1 expression also varied between the MD and vehicle control (Vehicle) groups; therefore, comparisons between the compound-treated groups and the Vehicle group were emphasized. Following treatment with the four candidate compounds, C3AR1 protein expression showed varying degrees of numerical reduction relative to the Vehicle group. Among them, the SM2-treated group exhibited the largest numerical reduction in C3AR1 protein expression, while SM1 also showed a downward trend. SM3 and SM4 produced relatively smaller changes. Although the overall difference among groups did not reach statistical significance (*p* = 0.0816), these results indicated compound-dependent variation in C3AR1 protein expression.

To further evaluate the effects of the candidate compounds on *C3AR1* mRNA expression, two independent primer pairs were used to quantify *C3AR1* transcript levels by reverse transcription quantitative polymerase chain reaction (RT-qPCR) (Figure 9E,F). Compared with the NC group, TNF-α stimulation was accompanied by an increase in *C3AR1* mRNA expression in the MD group. Following treatment with the candidate compounds, numerical reductions in *C3AR1* mRNA expression were observed to varying extents. SM1 showed the largest numerical reduction among the four treatment groups, with *C3AR1* mRNA expression decreasing from approximately 1.70-fold in the MD group to 1.06-fold, approaching the level observed in the NC group. SM4 treatment decreased *C3AR1* mRNA expression to approximately 1.24-fold, whereas the corresponding levels in the SM2- and SM3-treated groups were approximately 1.60- and 1.51-fold, respectively. The statistical support for these changes differed between the two primer sets, with a significant overall group difference detected for primer pair 1 (*p* = 0.0306) but not for primer pair 2. Overall, the *C3AR1* expression results revealed compound-dependent changes at both the protein and mRNA levels, with SM1 showing a relatively consistent numerical reduction across these measurements.

As shown in Figure 9G,H, the expression levels of the inflammation-related genes *IL6* and *MMP3* were further examined to evaluate the anti-inflammatory effects of the candidate natural small molecules. Compared with the NC group, TNF-α stimulation markedly increased *IL6* and *MMP3* expression, consistent with the establishment of an inflammatory response in MH7A cells. Following treatment with the four candidate compounds, *IL6* and *MMP3* expression showed compound-dependent reductions, with statistically significant differences observed among the experimental groups (*p* < 0.05). Among the four candidates, SM1 showed a relatively consistent reduction in both *IL6* and *MMP3* expression, particularly for MMP3.

Collectively, the four candidate natural small molecules produced distinct effects on C3AR1 expression and inflammatory responses in TNF-α-stimulated MH7A cells. Among them, SM1 showed a relatively consistent reduction in the inflammatory markers IL6 and MMP3, accompanied by changes in C3AR1 expression. These findings supported the further investigation of SM1 among the four candidate compounds.

## 3. Discussion

In this study, we established a pathway-driven computational drug discovery framework integrating cross-cohort pathway identification, candidate-target prioritization, natural-product screening, molecular simulation, and experimental evaluation. Sixteen independent RA-related gene-expression datasets were included, with 15 cohorts used for model development and one reserved for independent external validation. Rather than relying primarily on individual gene-level alterations, our strategy characterized disease-associated changes at the pathway level and incorporated cohort-aware feature selection to account for heterogeneity among independent datasets. The dataset-grouped LODO–RFA–SEFE framework, together with cross-algorithm consensus analysis, enabled the identification of pathway features that were repeatedly selected across different cohort-held-out conditions and machine-learning algorithms. This design provided a more robust basis for subsequent biological prioritization and linked cross-cohort transcriptomic evidence with downstream target and compound screening. Although the CAT+ET model showed moderate discrimination in the internal validation set (ROC-AUC = 0.783, 95% CI: 0.731–0.836), its performance decreased in the independent external validation cohort (ROC-AUC = 0.610, 95% CI: 0.496–0.720), with the confidence interval including 0.5. This result indicates limited cross-cohort generalizability and highlights the potential influence of inter-cohort heterogeneity on model performance. Accordingly, the predictive model should be considered exploratory and requires further validation in larger and more heterogeneous independent cohorts.

RA is a complex autoimmune inflammatory disease involving abnormal immune activation, persistent inflammatory mediator release, synovial dysregulation, and progressive joint tissue destruction. Increasing evidence indicates that these pathological processes arise from coordinated perturbations across multiple interconnected biological pathways rather than from a single molecular abnormality [28,29]. Consistent with this concept, 10 stable RA-associated pathways were identified in the present study, including the Notch signaling pathway, Complement and coagulation cascades, IL-17 signaling pathway, p53 signaling pathway, and Wnt signaling pathway. The involvement of pathways associated with immune-inflammatory regulation and cellular responses further reflects the molecular complexity of RA. Among these pathways, the Notch signaling pathway showed the highest global importance in the SHAP analysis, whereas Complement and coagulation cascades also exhibited high predictive importance together with strong cross-cohort selection stability. Considering its predictive contribution, cross-cohort stability, characteristic nonlinear association with model output, and biological relevance to immune-inflammatory processes, Complement and coagulation cascades was carried forward for subsequent target-level investigation.

It is noteworthy that the complement system, traditionally recognized for its role in pathogen elimination and immune defense, is also increasingly implicated in chronic inflammation and tissue injury in RA. Previous studies have demonstrated sustained activation of the complement cascade in RA synovial tissues, which has been associated with disease activity [30,31]. During complement activation, the generation of the anaphylatoxins C3a and C5a represents an important mechanism linking complement activation to inflammatory responses. Through their respective receptors, C3aR and C5aR, these complement-derived mediators can promote the recruitment and activation of immune cells, including macrophages, neutrophils, and T cells, and enhance the production of inflammatory cytokines such as TNF-α, IL1β, and IL6, thereby amplifying local inflammation and contributing to synovial pathology and joint tissue damage [32,33]. These findings provide a biological basis for the involvement of complement-associated signaling in RA and further support investigation of receptor-mediated complement signaling in the context of RA inflammation.

Within the Complement and coagulation cascades pathway, REMA identified several genes with relatively strong cross-cohort evidence, including CR1, CFB, ITGB2, C7, and C3AR1. Notably, C3AR1 was not the highest-ranked gene according to the REMA score, ranking below CR1, CFB, ITGB2, and C7. Its prioritization therefore did not rely on a single statistical ranking. Instead, the subsequent target-prioritization process integrated cross-cohort expression evidence with PPI relationships, MCODE network-module information, and existing biological evidence. Within this integrated framework, C3AR1 was positioned at the interface between complement-associated signaling and immune-inflammatory interactions, providing a biological rationale for carrying it forward as a candidate target for subsequent structure-based screening. This prioritization should therefore be interpreted as a multilevel evidence-integration step rather than as evidence that C3AR1 represents the highest-ranked or experimentally validated therapeutic target.

The REMA further provided a cross-cohort assessment of gene-expression alterations within the prioritized pathway. Considerable heterogeneity in expression patterns was observed across the included GEO cohorts, emphasizing the potential limitations of drawing conclusions from a single dataset. By integrating effect estimates across independent cohorts using a random-effects model, the analysis accounted for between-study heterogeneity while identifying expression patterns that were supported across datasets. In this context, the cross-cohort evidence for C3AR1 complemented, rather than independently determined, its subsequent prioritization. Together with the PPI, MCODE, and biological evidence, these results provided a convergent basis for advancing C3AR1 to downstream computational investigation. Nevertheless, although this prioritization was supported by multiple complementary analyses, the final integration of these evidence streams was not based on a fully prespecified quantitative decision rule. Therefore, a degree of post-hoc judgment remained in the final target selection and should be acknowledged as a limitation. Future studies incorporating prespecified and quantitative target-prioritization criteria may further improve the reproducibility of this framework.

Regarding natural-product discovery, we further established a large-scale virtual-screening workflow based on the COCONUT database, comprising approximately 470,000 natural-product compounds. Natural products represent an important source of structurally diverse bioactive molecules and continue to provide valuable chemical space for drug discovery [23,24]. Through sequential virtual screening, refined molecular docking, and subsequent structural evaluation, four candidate compounds (SM1–SM4) were prioritized for further investigation. Because molecular size contributed to the raw docking scores, ligand-efficiency normalization and multiple scoring approaches were further incorporated to complement the initial score-based ranking. In addition, self-redocking of JR14A yielded a top-pose RMSD of 3.88 Å, indicating limited recovery of the crystallographic binding pose and uncertainty in docking-based pose ranking. Therefore, the predicted binding orientations and residue-level interactions of SM1–SM4 should be interpreted cautiously rather than regarded as experimentally validated binding modes. In addition, PDB 8ZWF used in this study represents a JR14A-bound, BRIL-engineered active-state conformation of C3aR, with JR14A characterized as a C3aR agonist. Therefore, the predicted compatibility of SM1–SM4 with this binding region should not be interpreted as evidence of antagonistic or inhibitory activity, and molecular docking alone cannot determine their specific pharmacological modes of action. Further receptor-binding and functional assays are required to validate their direct interactions with C3AR1 and the corresponding pharmacological effects. The selected compounds were subsequently evaluated by MD simulations in an explicit lipid-bilayer environment, providing additional information on their receptor-associated structural, dynamic, and energetic characteristics. Notably, several residues, including Arg143, Tyr156, and Arg165, recurred in both the docking interaction analysis and MD-based residue-level analyses. However, given the limited pose recovery observed in JR14A self-redocking, these recurrent computational features should be regarded as hypotheses for subsequent experimental investigation rather than evidence of specific receptor–ligand contacts.

Based on the computational prioritization, four candidate natural compounds (SM1–SM4) were further evaluated in a TNF-α-induced MH7A synoviocyte inflammatory model. MH7A cells are widely used as a human RA fibroblast-like synoviocyte model for investigating inflammatory responses and evaluating candidate anti-inflammatory compounds [34]. TNF-α, a major pro-inflammatory cytokine involved in RA pathogenesis, induces inflammatory responses in synoviocytes, including increased expression of IL6 and MMP3. In the present study, the candidate compounds produced compound-dependent changes in these inflammatory readouts, with SM1 showing a relatively consistent reduction in IL6 and MMP3. Given the roles of IL6 in inflammatory amplification and MMP3 in extracellular-matrix degradation and synovial tissue damage, these findings support the anti-inflammatory activity of SM1 in the TNF-α-stimulated MH7A model.

We further examined whether these cellular effects were accompanied by changes in the computationally prioritized candidate target C3AR1. C3AR1 mediates cellular responses to the complement-derived anaphylatoxin C3a and has been implicated in immune-inflammatory processes [35]. Directional changes in C3AR1 protein expression were observed following treatment with the candidate compounds, with SM2 showing a numerically more pronounced reduction; however, the overall difference among groups did not reach statistical significance (*p* = 0.0816), and no pairwise comparisons were statistically significant. *C3AR1* mRNA expression was additionally examined using two independent primer pairs, but the transcriptional responses were modest, and no pairwise comparisons reached statistical significance after multiple-comparison adjustment. These expression patterns suggest a potential compound-associated trend in C3AR1-related readouts; however, the current evidence remains insufficient to establish significant C3AR1 regulation, direct compound–C3AR1 engagement, or a C3AR1-dependent mechanism. Further studies with larger sample sizes and direct functional validation are needed to clarify this relationship.

Among the four candidates, SM1 showed a relatively consistent reduction in the inflammatory markers IL6 and MMP3, accompanied by changes in C3AR1 expression. Together with its favorable computational characteristics identified through molecular docking and membrane MD simulations, these cellular findings provide convergent support for prioritizing SM1 for further investigation. The distinct response patterns observed across the four candidate compounds further suggest compound-dependent differences in their cellular activities and provide additional information for subsequent mechanistic characterization. The SwissADME and ADMETlab 3.0 predictions further provided complementary information on the physicochemical, drug-likeness, and ADMET characteristics of SM1–SM4. Although several potentially favorable properties were identified, the predictions also indicated compound-specific developability and safety-related limitations. These findings highlight the preliminary nature of the in silico ADMET assessment and the need for subsequent experimental pharmacokinetic and toxicological characterization. Taken together, the integration of computational prioritization with cellular inflammatory phenotypes and molecular-expression measurements supports the prioritization of SM1 as a candidate warranting further investigation. However, direct target-engagement and functional studies, together with experimental pharmacokinetic and safety evaluation, will be required to establish its pharmacological potential.

## 4. Materials and Methods

### 4.1. Data Collection and Preprocessing

The 16 RA-related gene expression datasets were preprocessed using the GEOquery package (version 2.72.0) and limma package (version 3.60.6) implemented in the R environment (version 4.4.0). The preprocessing procedures included gene annotation, missing value handling, and data normalization. Subsequently, all human KEGG pathway information was downloaded from the KEGG database using the clusterProfiler package (version 4.12.6), and a pathway–gene annotation dataset was constructed. In total, 359 KEGG pathways and 8842 associated genes were obtained.

GSVA was performed using the GSVA package (version 1.52.3) to quantify KEGG pathway activity in each of the 16 RA-related gene expression datasets. Pathway enrichment scores were calculated for each sample across the selected KEGG pathways using a Gaussian kernel (kcdf = “Gaussian”). Subsequently, pathway enrichment scores from all datasets were integrated to construct the final sample–pathway matrix ($A_{Sample\times Pathway}$), in which rows represented RA patients or healthy controls, columns represented KEGG pathways, and matrix elements corresponded to the GSVA enrichment scores of individual pathways.

### 4.2. Identification of Key Pathways Using a Combined RFA–SEFE Strategy

To identify RA-associated pathways with robust cross-cohort stability, we developed a two-stage feature-selection strategy integrating RFA and SEFE within a dataset-grouped LODO framework. Seven ensemble learning algorithms were independently employed for feature selection and evaluation: RF, ET, GBT, HGB, LGB, XGB, and CAT.

In the outer LODO procedure, one GEO dataset was completely held out as the held-out cohort in each iteration, while the remaining datasets collectively constituted the outer-training set. This procedure was repeated until each development cohort had served once as the held-out cohort. Within each outer-training set, GroupKFold cross-validation was performed using the GEO dataset identifier as the grouping variable. Five folds were used by default; when fewer than five training cohorts were available, the number of folds was adjusted to the number of available cohorts. This dataset-level partitioning ensured that samples originating from the same GEO cohort were never simultaneously assigned to the training and validation folds, thereby reducing the risk of information leakage arising from cohort-specific characteristics.

For each learning algorithm, hyperparameters were first optimized across the full set of candidate pathways using grid search, with the mean ROC-AUC across the inner GroupKFold folds as the primary optimization criterion. The model was then fitted using the optimal hyperparameters, and pathway features were ranked in descending order according to their feature importance.

During the RFA stage, nested candidate feature subsets containing the top 1 through top k ranked pathways were sequentially constructed. Each candidate subset was evaluated by GroupKFold cross-validation, and the subset achieving the highest mean ROC-AUC was retained. When multiple subsets yielded identical mean ROC-AUC values, the subset containing fewer features was preferentially selected to favor model parsimony.

SEFE was subsequently applied to the candidate feature set retained by RFA. Feature importance was recalculated, and features were sequentially considered for removal in ascending order of importance. After each candidate feature was removed, model performance was reassessed using GroupKFold cross-validation. A removal was accepted only when the resulting mean ROC-AUC was strictly higher than the current best value; otherwise, the feature was retained. This procedure continued until all candidate features had been evaluated or only a single feature remained, yielding the final pathway subset for that LODO fold.

Following the two-stage RFA–SEFE procedure, hyperparameters were re-optimized using the final retained feature subset, and the model was refitted on the entire outer-training set. The corresponding held-out GEO cohort remained completely excluded from feature selection and hyperparameter optimization and was used only once for independent evaluation of the model generated in that fold, thereby yielding cohort-held-out predictions.

To quantify the cross-cohort stability of pathway selection, the pathways retained in each LODO fold were recorded, and the selection frequency of each pathway was calculated as the number of folds in which it was retained divided by the total number of LODO folds. After completion of all LODO iterations, the GroupKFold-based hyperparameter optimization, RFA, and SEFE procedures were repeated using the complete development dataset. Hyperparameters were then re-optimized on the final retained features to obtain the pathway feature set and corresponding model parameters for each learning algorithm. These results were subsequently used to define the key pathway features for downstream predictive modeling.

### 4.3. Construction of Predictive Models Using Ensemble Learning Strategies

After completion of pathway selection, predictive models were reconstructed using the final set of key pathway features. This model-development stage was conducted separately from the pathway-selection procedure detailed in Section 4.2, with the selected pathway features fixed before subsequent model training and comparison.

The seven base classifiers described above were retrained using the selected pathway features. For each classifier, hyperparameters were optimized by grid search combined with stratified five-fold cross-validation, using the mean cross-validated ROC-AUC as the primary optimization criterion. After the optimal hyperparameters had been identified, each base classifier was refitted on the complete training set. Their predicted probabilities were subsequently used to construct Stacking and Soft Voting ensemble models.

To systematically evaluate combinations of the seven base classifiers, all non-empty subsets of the seven classifiers were considered, resulting in $2^{7}-1=127$ candidate model configurations. These comprised seven individual base classifiers and 120 Stacking ensembles containing at least two base learners.

To reduce overfitting during Stacking model development, stratified five-fold cross-validation was used to generate OOF predictions within the training set. Specifically, for each cross-validation fold, the constituent base learners were fitted exclusively on the corresponding training subset and used to generate predicted probabilities for the held-out validation subset. Predictions from all validation folds were then concatenated to generate complete OOF predictions, which served as input features for the meta-learner. The candidate models were evaluated and ranked primarily according to the ROC-AUC derived from their OOF predictions, with PR-AUC additionally examined to characterize discriminative performance in the presence of potential class imbalance. The eight highest-performing Stacking models were retained and subsequently refitted using the complete training set.

In parallel, a Soft Voting ensemble incorporating all seven base classifiers was constructed. The final predicted probability of the Soft Voting model was obtained by averaging the predicted probabilities generated by the individual base classifiers. Its OOF performance was likewise evaluated using stratified five-fold cross-validation. The Soft Voting model was not included in the initial ranking of Stacking configurations but was retained as an independent ensemble strategy for subsequent comparison.

The final candidate model set therefore comprised 16 models: seven individual base classifiers, eight selected Stacking ensembles, and one Soft Voting ensemble. All 16 candidate models were subsequently evaluated in an internal validation set that was not used for model training or hyperparameter optimization and in an independent external validation set. Neither the internal validation set nor the external validation set contributed to model fitting or hyperparameter optimization, allowing assessment of predictive performance in the internal validation data and generalizability in independent external data.

### 4.4. Model Training and Performance Evaluation

Model performance was comprehensively evaluated using eight metrics: ROC-AUC, PR-AUC, accuracy, F1 score, PPV, NPV, sensitivity, and specificity. Because potential class imbalance between RA and healthy control samples could affect the interpretation of ROC-AUC, PR-AUC was additionally reported as a complementary measure of discriminative performance. RA was defined as the positive class; accordingly, sensitivity represented the ability of the model to correctly identify RA samples, whereas specificity represented its ability to correctly identify healthy controls.

To avoid selecting the final model on the basis of a single performance metric, TOPSIS was used to integrate the eight performance measures. TOPSIS analyses were performed separately for the internal validation set and the independent external validation set. ROC-AUC, PR-AUC, accuracy, F1 score, PPV, NPV, sensitivity, and specificity were all treated as benefit criteria, such that higher values represented better predictive performance.

The 16 candidate models were independently ranked according to their TOPSIS scores in the internal validation set and the external validation set, and the three highest-ranked models from each evaluation dataset were retained. The two Top-3 model sets were then intersected, and the model appearing among the three highest-ranked candidates in both the internal validation set and the independent external validation set was selected as the final predictive model. This selection strategy was designed to jointly consider predictive performance in the internal validation data and generalizability to independent external data, while reducing reliance on any single dataset or individual performance metric.

To quantify uncertainty in model performance estimates, nonparametric bootstrap resampling was performed separately in the internal validation set and the independent external validation set to calculate 95% confidence intervals (CIs) for the performance metrics. For each evaluation dataset, 2000 bootstrap samples were generated by sampling with replacement, with each resampled dataset containing the same number of observations as the original dataset. Models were not retrained during bootstrap resampling; instead, performance metrics were recalculated from the observed class labels and the predictions generated by the previously fitted models. Bootstrap iterations containing samples from only one class were excluded. The 2.5th and 97.5th percentiles of the resulting bootstrap distributions were used as the lower and upper bounds of the 95% CIs, respectively.

### 4.5. Model Interpretability Analysis

To characterize the decision-making process of the final predictive model and quantify the contribution of individual pathways to model predictions, SHAP analysis was performed. Pathway features from the training data were used as the background data for model interpretation, and SHAP values were calculated for individual samples in the internal validation set and the independent external validation set to quantify the magnitude and direction of each pathway’s contribution to the model output.

Global feature importance was quantified using the mean absolute SHAP value for each pathway. SHAP importance plots were used to summarize the overall ranking of pathway contributions, whereas SHAP beeswarm plots were generated to visualize the relationships among pathway activity levels, SHAP values, and the direction of their effects on model predictions.

For the highest-ranking pathways, SHAP dependence plots were further generated to examine the relationship between pathway activity and SHAP values and to characterize potential nonlinear associations between pathway activity and model output. SHAP patterns were additionally compared between the internal validation set and the independent external validation set to assess the consistency and interpretative stability of the contributions of the major pathways across independent evaluation datasets.

### 4.6. Identification of Potential Therapeutic Targets Based on Random-Effects Meta-Analysis

For target-level prioritization within the pathway selected for downstream investigation, REMA was used to evaluate and prioritize genes within the pathway.

First, differential expression analysis was independently performed for each gene expression dataset. For each gene, the corresponding effect size and statistical value were extracted from each dataset, and the standard error was calculated based on the statistical value:

$$SE_{i}=\frac{|logFC_{i}|}{|t_{i}|}$$

where i represents the individual dataset $\left(i=1,2,\cdots ,16\right)$.

Subsequently, the effect size obtained from each dataset was used as the estimated effect, while the corresponding standard error was incorporated as weighting information. A random-effects model (REM) was applied to perform meta-analysis across multiple datasets, generating the combined effect size ($logFC_{RE}$), 95% confidence interval, and heterogeneity index ($H^{2}$) for each gene:

$$y_{i}=\mu +u_{i}+\epsilon_{i}$$

where $\epsilon_{i}∼N(0,SE_{i}^{2})$ represents the observation error, $u_{i}∼N(0,\tau^{2})$ represents the between-study variance, and $\tau^{2}$ denotes the variance among different studies. The total variance was calculated as:

$$Var(y_{i})=SE_{i}^{2}+\tau^{2}$$

and the corresponding weight was defined as:

$$w_{i}=\frac{1}{SE_{i}^{2}+\tau^{2}}$$

The pooled effect size was subsequently calculated as:

$$logFC_{RE}=\frac{\sum_{i=1}^{k}w_{i}y_{i}}{\sum_{i=1}^{k}w_{i}}$$

where k represents the number of included studies.

Finally, to prioritize candidate genes, a comprehensive scoring system (*Score*) was established:

$$Score=\frac{|logFC_{RE}|\times \sqrt{k}}{\sqrt{1+\frac{H^{2}}{100}}}$$

Accordingly, genes within the identified key pathways were ranked in descending order according to their *Score* values, and genes with higher scores were considered potential therapeutic target candidates for RA.

### 4.7. Construction of the Molecular Virtual Library

In this study, approximately 600,000 natural small molecules from the COCONUT database (version 2025-06) were utilized for potential therapeutic compound screening. First, the complete SDF file was downloaded from the COCONUT database, and individual molecular structures were extracted using RDKit (version 2025.03.5). Each input molecule was subsequently subjected to a series of preprocessing procedures.

The preprocessing workflow mainly consisted of the following steps. First, molecular filtering based on the number of heavy atoms was performed. Each molecule was required to contain at least 10 heavy atoms to ensure sufficient structural complexity and to exclude overly simple compounds from subsequent screening procedures. Meanwhile, to avoid excessive computational costs caused by large molecular structures during optimization, the total number of atoms per molecule was restricted to no more than 300 atoms. Furthermore, only molecules represented as single-fragment structures were retained to ensure that molecular optimization was performed on complete compounds rather than disconnected molecular fragments.

For molecules lacking three-dimensional (3D) conformations, reasonable 3D structures were generated using the Experimental-Torsion Knowledge Distance Geometry (ETKDG) method implemented in RDKit. Subsequently, molecular geometry optimization was performed using the Merck Molecular Force Field (MMFF), an empirical small-molecule force field widely applied for molecular structure optimization and energy minimization. If MMFF optimization failed, the Universal Force Field (UFF) method was alternatively applied. Due to its broader applicability, UFF is particularly suitable for molecular structures that are difficult to optimize using MMFF and generally provides stable optimized conformations. Finally, successfully optimized molecules were converted into PDBQT format and stored for subsequent molecular docking.

### 4.8. Molecular Docking Screening

Following target prioritization, the three-dimensional structure of the candidate receptor was obtained and prepared for molecular docking. Missing side-chain residues and loop regions were repaired using Modeller (version 9.14). Crystallographic water molecules, the original ligand, and unnecessary co-crystallized components were subsequently removed using PyMOL (version 1.8.0). Hydrogen atoms and charges were assigned using AutoDockTools (version 1.5.7), and the prepared receptor was converted to PDBQT format. The docking center was defined according to the region occupied by the experimentally resolved ligand, and a docking search space of 30 × 30 × 30 Å^3^ was established for subsequent structure-based virtual screening.

Large-scale preliminary virtual screening of the natural-product library was first performed using Vina-GPU 2.1, with the thread parameter set to 8000 and the remaining parameters maintained at their default settings. Compounds were ranked according to their docking scores, and the top 500 compounds from the preliminary screening were subsequently subjected to refined semi-flexible molecular docking using AutoDock Vina 1.1.2. Three independent docking runs were performed for each compound, and the mean and standard deviation (mean ± SD) of the three docking scores were calculated for subsequent ranking and analysis. Based on the mean Vina 1.1.2 docking score, the top 100 compounds were retained as the candidate set for further evaluation.

Because raw docking scores may be influenced by ligand size and scoring-function characteristics, the number of heavy atoms in each ligand was calculated, and Spearman rank correlation analysis was performed to evaluate the association between ligand heavy-atom count and docking score. This analysis was conducted separately for all 500 natural-product compounds subjected to refined docking and for the Top-100 subset selected according to Vina 1.1.2. Ligand efficiency (LE) was additionally calculated as a complementary size-normalized metric according to:

$$LE=-\frac{S}{N_{HA}}$$

where S represents the mean docking score (kcal/mol) obtained from three independent docking runs using the corresponding scoring approach and $N_{HA}$ represents the number of ligand heavy atoms. LE values were calculated separately from the mean docking scores obtained using Vina 1.1.2, Vina 1.2.7, and Vinardo. LE was used solely as a relative size-normalized metric and was not interpreted as an estimate of experimental binding affinity.

To reduce reliance on a single scoring approach, the Top-100 compounds selected according to Vina 1.1.2 were additionally evaluated using Vina 1.2.7 and Vinardo. Three independent calculations were likewise performed for each compound using Vina 1.2.7 and Vinardo, and the corresponding mean docking scores and standard deviations were calculated. Because the numerical scales of raw scores generated by different scoring approaches are not necessarily directly comparable, a rank-based consensus strategy was applied rather than directly averaging the raw docking scores. For each scoring approach, the Top-100 compounds were independently ranked from the most to the least favorable mean docking score, with rank 1 assigned to the most favorable score. The arithmetic mean of the three individual ranks was then defined as the consensus rank score:

$$R_{consensus}=\frac{R_{Vina1.1.2}+R_{Vina1.2.7}+R_{Vinardo}}{3}$$

where $R_{Vina1.1.2}$, $R_{Vina1.2.7}$ and $R_{Vinardo}$ represent the ranks of the same compound obtained using the three respective scoring approaches. A lower consensus rank score indicated a more favorable overall relative ranking across the scoring approaches. This consensus analysis was used to assess cross-method ranking consistency rather than to redefine the initial Top-100 candidate set selected according to Vina 1.1.2.

Finally, the predicted binding poses of the Top-100 candidates were subjected to qualitative visual inspection to assess plausible accommodation within the predefined receptor-binding region and reasonable ligand–receptor interaction geometry. This step served as a structural plausibility check to identify evidently implausible predicted poses rather than as an additional quantitative reranking procedure. Compounds passing this assessment were further considered according to their practical availability, and the final candidate compounds were selected for subsequent molecular dynamics simulations and experimental evaluation. The physicochemical properties, drug-likeness, and ADMET profiles of the final candidate compounds were further predicted using SwissADME and ADMETlab 3.0. Representative physicochemical and drug-likeness parameters, together with absorption, distribution, metabolism, excretion, and toxicity-related properties, were evaluated to provide complementary in silico characterization of the selected compounds.

### 4.9. Molecular Dynamics Simulation

To account for the membrane-associated nature of the target receptor, MD simulations of the protein–ligand complexes were performed in an explicit lipid bilayer environment. The membrane-embedded receptor system was constructed based on the protein structure used for molecular docking, and the selected ligand-binding conformations were used as the initial poses for subsequent simulations. The membrane systems were prepared using CHARMM-GUI and subsequently converted into AMBER-compatible simulation systems. The protein was parameterized using the AMBER ff14SB force field, whereas the membrane lipids were described using the Lipid21 force field.

For each ligand, restrained electrostatic potential (RESP) atomic charges were derived from quantum-chemical calculations performed using Gaussian 16W at the HF/6-31G* level. Ligand force-field parameters were generated using the Antechamber and Parmchk2 modules of AmberTools with the GAFF2 force field. Ligand coordinates were aligned to the equilibrated membrane-embedded receptor, and the corresponding protein–ligand complexes were subsequently assembled using the tleap module. An identical ligand-parameterization and system-construction workflow was applied to all simulated systems to ensure methodological consistency in subsequent comparative analyses.

Each membrane-embedded complex was explicitly solvated using the TIP3P water model with approximately 12 Å of solvent padding. Sodium and chloride ions were introduced to neutralize the net charge of each system and establish an ionic strength of approximately 0.15 M NaCl. Periodic boundary conditions were applied throughout the simulations. Long-range electrostatic interactions were calculated using the particle-mesh Ewald (PME) method with a 10 Å nonbonded cutoff. Covalent bonds involving hydrogen atoms were constrained using the SHAKE algorithm, permitting a simulation time step of 2 fs.

Each system was first subjected to 10,000 steps of energy minimization, consisting of 5000 steps of steepest-descent minimization followed by 5000 steps of conjugate-gradient minimization, with positional restraints of 10 kcal mol^−1^ Å^−2^ applied to the protein backbone and ligand. The minimized systems were subsequently heated gradually from 0 to 310 K over 100 ps under the NVT ensemble while maintaining the same positional restraints. The systems were then equilibrated under the NPT ensemble at 310 K and 1 atm through a stepwise restraint-release protocol. Specifically, positional restraint strengths of 5, 2, and 1 kcal mol^−1^ Å^−2^ were successively applied for 0.5, 0.5, and 1 ns, respectively, followed by an additional 2 ns of unrestrained NPT equilibration.

Following equilibration, each protein–ligand complex was subjected to a 100-ns unrestrained production MD simulation under the NPT ensemble at 310 K and 1 atm. The production simulation was divided into ten consecutive 10-ns segments to facilitate trajectory restart and management, with each segment initiated directly from the final coordinates and velocities of the preceding segment. Coordinates were recorded every 10 ps. All simulations were performed using the GPU-accelerated pmemd.cuda engine implemented in Amber 22.

The resulting trajectories were processed and analyzed using Cpptraj in AmberTools after correction of periodic-boundary artifacts. Structural stability and dynamic behavior of the protein–ligand complexes were evaluated using RMSD, RMSF, Rg, SASA, and intermolecular hydrogen-bond analyses. PCA was further performed to characterize the dominant collective motions sampled during the simulations. The first two principal components (PC1 and PC2) were used as reaction coordinates to construct the FEL. The Gibbs free energy was calculated according to the Boltzmann distribution:

$$\Delta G=-k_{B}\cdot T\cdot ln\left(\frac{P}{P_{max}}\right)$$

where $k_{B}$ represents the Boltzmann constant, T represents the simulation temperature, P represents the probability density of a specific conformational state, and $P_{max}$ represents the maximum probability density observed within the system. DCCM analysis was additionally conducted to characterize correlated and anticorrelated motions among protein residues.

Binding free energies were further estimated from the production trajectories using both MM/GBSA and MM/PBSA approaches. For both calculations, trajectory frames 2001–10,000 were sampled at an interval of 100 frames, yielding 80 snapshots for each protein–ligand system. Given that coordinates were recorded every 10 ps, the sampled frames corresponded approximately to the 20–99 ns portion of each 100-ns production trajectory. An identical frame-selection scheme was applied to all systems to ensure consistency in comparative binding-energy analyses.

For the MM/GBSA calculations, the generalized Born model was applied using igb = 5 with an ionic strength of 0.150 M. The nonpolar solvation contribution was calculated using a surface-tension coefficient of 0.0072 kcal mol^−1^ Å^−2^. For the MM/PBSA calculations, the internal and external dielectric constants were set to 1.0 and 80.0, respectively, with an ionic strength of 0.150 M and a solvent probe radius of 1.4 Å. The nonpolar solvation contribution was treated using the solvent-accessible surface-area-based model. Entropic contributions were not included in either the MM/GBSA or MM/PBSA calculations. Per-residue free-energy decomposition was additionally performed for both approaches to characterize the energetic contributions of individual protein residues to ligand binding.

### 4.10. In Vitro Experimental Evaluation

For experimental evaluation of the computationally prioritized candidate compounds, a TNF-α-induced MH7A fibroblast-like synoviocyte inflammatory model was established to evaluate the in vitro pharmacological activities of the selected candidate natural small molecules.

First, the effect of candidate compounds on MH7A cell viability was assessed using the CCK-8 assay, and appropriate working concentrations were determined for subsequent functional experiments. Subsequently, a therapeutic intervention model was established to evaluate the anti-inflammatory activities of candidate compounds. The experimental groups included the NC, MD, Vehicle, and candidate-compound treatment groups. In the Vehicle group, TNF-α-stimulated cells were treated with dimethyl sulfoxide (DMSO) at the same final concentration used for candidate compound treatment groups.

Subsequently, the mRNA expression levels of inflammation-associated factors (IL6 and MMP3) and candidate target genes were determined using RT-qPCR. The expression levels of target proteins were further evaluated using Western blot analysis to examine whether treatment with the candidate natural small molecules was accompanied by changes in target expression.

Detailed experimental procedures, reagent preparation, experimental conditions, detection methods, and statistical analysis procedures are described in the Supplementary Methods S9. Collectively, these in vitro experiments were performed to validate the anti-inflammatory activity of candidate natural small molecules and further evaluate their regulatory effects on potential therapeutic targets, thereby providing experimental support for the reliability of the computational drug discovery findings.

## 5. Conclusions

In conclusion, this study established a pathway-driven computational drug discovery framework integrating cross-cohort pathway identification, candidate-target prioritization, natural-product virtual screening, molecular dynamics simulations, and in vitro evaluation. However, the reduced performance observed in the independent external validation set indicates limited cross-cohort generalizability, and the predictive model should therefore be considered exploratory pending further validation in larger and more heterogeneous independent cohorts. Complement and coagulation cascades was prioritized for downstream investigation, and integrated cross-cohort expression, network, and biological evidence further prioritized C3AR1 as a candidate target. Four natural compounds (SM1–SM4) were subsequently advanced for computational and experimental evaluation, among which SM1 showed comparatively favorable computational characteristics and a relatively consistent reduction in the inflammatory markers IL6 and MMP3 in TNF-α-stimulated MH7A cells. C3AR1 expression showed a non-significant compound-associated trend, warranting further investigation. Together, these computational and experimental findings support SM1 as a promising candidate for further pharmacological and mechanistic investigation. Further studies directly assessing compound–C3AR1 engagement and receptor function will be required to clarify the pharmacological relationship between SM1 and C3AR1. Overall, this framework provides a systematic strategy for connecting robust cross-cohort RA pathway features with candidate targets and natural compounds and offers a foundation for subsequent natural-product drug discovery in RA.

## Figures and Tables

**Table 1:** Characteristics and analytical roles of the GEO datasets used for model development and external validation.

GEO Dataset	Platform	Total Samples in GEO	Included HC	Included RA	Included Total	Tissue Source	Role in Revised Analysis
GSE1919	GPL91	15	5	5	10	Synovial tissue	Development
GSE12021	GPL96 & GPL97	57	9	12	21	Synovial tissues	Development
GSE17755	GPL1291	244	53	112	165	Peripheral blood	Development
GSE23561	GPL10775	35	9	6	15	Peripheral blood	Development
GSE29746	GPL4133	31	11	9	20	Synovial fibroblasts	Development
GSE45291	GPL13158	805	20	493	513	Whole blood	Development
GSE55235	GPL96	30	10	10	20	Synovial tissues	Development
GSE55457	GPL96	33	10	13	23	Synovial tissues	Development
GSE56649	GPL2509	22	9	13	22	Peripheral blood	Development
GSE68689	GPL2513	21	5	16	21	Whole blood	Development
GSE77298	GPL2516	23	7	16	23	Synovial tissue	Development
GSE89408	GPL11154	218	28	152	180	Synovial biopsies	Development
GSE93272	GPL570	275	43	232	275	Whole blood	Development
GSE110169	GPL13667	243	77	84	161	Whole blood	Development
GSE121894	GPL25734	58	11	18	29	Peripheral blood	Development
Development total	—	—	307	1191	1498	—	—
GSE117769	GPL16791	120	50	51	101	Peripheral blood	Independent external validation
Overall included	—	—	357	1242	1599	—	—

Note: “Total samples in GEO” refers to the total number of samples deposited in each GEO Series, whereas “Included HC,” “Included RA,” and “Included total” refer to the samples retained for the RA-versus-healthy-control analysis. Fifteen GEO datasets comprising 1498 samples (1191 RA and 307 HC) were used for model development, while GSE117769 comprising 101 samples (51 RA and 50 HC) was reserved exclusively for independent external validation in the revised analysis. HC, healthy control; RA, rheumatoid arthritis.

**Table 2:** Basic information and docking scores of the four candidate natural compounds and the structure-derived reference ligand JR14A obtained using different scoring approaches.

ID	Structure	Name	Formula	COCONUT ID	PubChem CID	Vina 1.1.2 Docking Score (kcal/mol)	Vina 1.2.7 Docking Score (kcal/mol)	Vinardo Docking Score (kcal/mol)
SM1	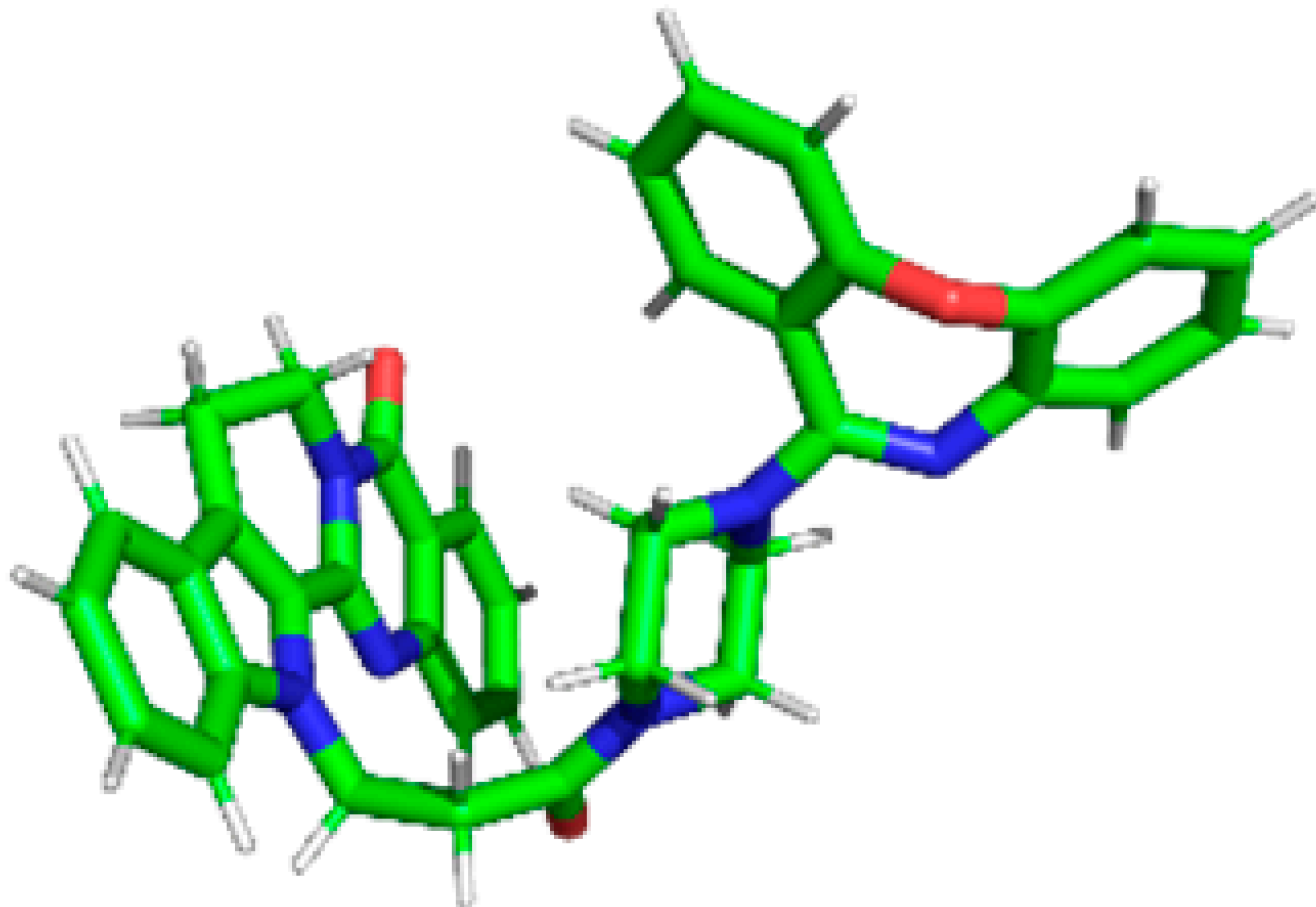	13-{3-[4-(dibenzo[b,f][1,4]oxazepin-11-yl)piperazin-1-yl]-3-oxopropyl}-8,13-dihydroindolo[2’,3’:3,4]pyrido[2,1-b]quinazolin-5(7H)-one	C38H32N6O3	CNP0305766.0	71755006	−12.40 ± 0.00	−12.43 ± 0.03	−7.41 ± 0.01
SM2	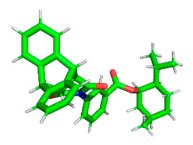	[(1~{R},2~{R},5~{R})-2-isopropyl-5-methyl-cyclohexyl] 3-[(15~{R},19~{R})-16,18-dioxo-17-azapentacyclo[6.6.5.0^{2,7}.0^{9,14}.0^{15,19}]nonadeca-2,4,6,9,11,13-hexaen-17-yl]benzoate	C35H35NO4	CNP0580826.2	7121046	−12.40 ± 0.00	−11.61 ± 0.01	−7.67 ± 0.32
SM3	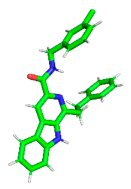	(7S)-N-((S)-1-((4-methylbenzyl)amino)-1-oxo-3-phenylpropan-2-yl)-5-oxo-7,8,13,13b-tetrahydro-5H-benzo[1,2]indolizino[8,7-b]indole-7-carboxamide	C36H32N4O3	CNP0542742.1	16405311	−12.33 ± 0.06	−10.16 ± 0.40	−5.97 ± 0.70
SM4	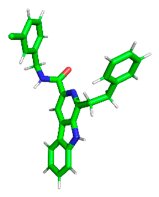	N-(3-chlorobenzyl)-1-(2-phenylethyl)-9H-beta-carboline-3-carboxamide	C27H22ClN3O	CNP0383412.0	5576003	−12.20 ± 0.00	−10.53 ± 0.16	−7.33 ± 0.22
JR14A	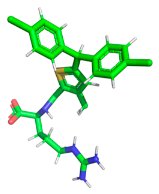	(2~{S})-5-[bis(azanyl)methylideneamino]-2-[[5-[bis(4-chlorophenyl)methyl]-3-methyl-thiophen-2-yl]carbonylamino]pentanoic acid	C25H26Cl2N4O3S	—	145996525	−8.67 ± 0.06	−8.34 ± 0.72	−5.44 ± 0.10

Note: Docking scores are presented as the mean ± SD of three independent docking runs for each scoring approach. JR14A is the structure-derived reference ligand from the 8ZWF receptor structure and was not included in the natural-product candidate ranking. “—” indicates not applicable.

## Data Availability

The datasets generated and/or analyzed during the current study are available in the Supplementary Materials. Processed datasets and source code supporting the findings of this study are available from the corresponding author upon request.

## Supplementary Materials

The following supporting information can be downloaded at: https://www.mdpi.com/article/10.3390/ph19101651/s1, Supplementary Data S1. Human KEGG pathway dataset; Supplementary Data S2. GSVA enrichment score matrix of KEGG pathways; Supplementary Data S3. RA-associated KEGG pathways; Supplementary Data S4. Performance metrics of the candidate models in the internal test set and independent external validation set; Supplementary Data S5. PPI network of genes in the hsa04610 pathway; Supplementary Data S6. Target evaluation results for the hsa04610 pathway; Supplementary Data S7. Docking scores, ligand efficiencies, and consensus ranking of the Top 100 compounds; Supplementary Data S8. Selected predicted physicochemical, drug-likeness, and ADMET properties of SM1–SM4; Supplementary Methods S9. In vitro validation of candidate C3AR1-related compounds; Supplementary Data S10. Uncropped original Western blot images; Supplementary Data S11. NMR characterization of small molecules; Supplementary Data S12. Complete R session information.

## Abbreviations

ADMET, absorption, distribution, metabolism, excretion, and toxicity; CAT, CatBoost; DCCM, dynamic cross-correlation matrix; ET, Extra Trees; FEL, free-energy landscape; GBT, Gradient Boosting Trees; GEO, Gene Expression Omnibus; HGB, Histogram-based Gradient Boosting; LE, ligand efficiency; LGB, Light Gradient Boosting Machine; LODO, leave-one-dataset-out; MCODE, Molecular Complex Detection; MD, molecular dynamics; MM/GBSA, molecular mechanics/generalized Born surface area; MM/PBSA, molecular mechanics/Poisson–Boltzmann surface area; NPV, negative predictive value; OOF, out-of-fold; PCA, principal component analysis; PPI, protein–protein interaction; PPV, positive predictive value; PR, precision–recall; PR-AUC, area under the precision–recall curve; RA, rheumatoid arthritis; REMA, random-effects meta-analysis; RF, Random Forest; RFA, recursive feature addition; Rg, radius of gyration; RMSD, root mean square deviation; RMSF, root mean square fluctuation; ROC, receiver operating characteristic; ROC-AUC, area under the receiver operating characteristic curve; RT-qPCR, reverse transcription quantitative polymerase chain reaction; SASA, solvent-accessible surface area; SEFE, stepwise exhaustive feature elimination; SHAP, SHapley Additive exPlanations; ssGSEA, single-sample gene set enrichment analysis; TOPSIS, Technique for Order Preference by Similarity to Ideal Solution; XGB, Extreme Gradient Boosting.
