# Supplementary material for: A Pathway-Driven Computational Drug Discovery Framework Integrating Machine Learning and Virtual Screening for Identification of Anti-Rheumatoid Arthritis Natural Small Molecules
Source: Pharmaceuticals (Basel). 2026 Oct 19;19(10):1651. doi: 10.3390/ph19101651 (PMC13708292; doi:10.3390/ph19101651)
Supplement: Supplementary file 1 [file pharmaceuticals-19-01651-s001.zip › Supplementary_DAta_S09.pdf]

Supplementary Methods S9

In vitro validation of candidate *C3AR1*-related compounds

1. Cell culture

Human rheumatoid arthritis fibroblast-like synoviocytes (MH7A) were used as an in vitro inflammatory cell model. MH7A cells were purchased from AnWei Biotechnology (Shanghai, China) and corresponded to the RIKEN Cell Bank cell line RCB1512 (RRID: CVCL_0427). The cells were cultured in MH7A complete medium (Fuheng Biology, Shanghai, China) at 37°C in a humidified incubator containing 5% CO_2. Cells were passaged every 2–3 days, and cells between passages 3 and 9 were used for subsequent experiments.

2. Candidate compounds and reagent preparation

Four C3AR1-related candidate compounds identified through previous bioinformatics and network pharmacology analyses were selected and designated as compounds SM1–SM4. Stock solutions of all compounds were prepared in dimethyl sulfoxide (DMSO; Titan Scientific Co., Ltd., Shanghai, China) and stored at −20°C protected from light. Before experiments, the stock solutions were diluted with complete culture medium to the required working concentrations.

Recombinant human TNF-α (ABclonal, catalog no. RP00993, Wuhan, China) was dissolved in sterile phosphate-buffered saline (PBS) to prepare a 10 μg/mL stock solution, aliquoted, and stored at −80°C. The final concentration of TNF-α used for MH7A inflammatory stimulation was 20 ng/mL.

Table S1. Information of candidate compounds

Compound	Molecular formula	Molecular weight
SM1	$C_{38}H_{32}N_6O_3$	620.70
SM2	$C_{35}H_{35}NO_4$	533.66
SM3	$C_{36}H_{32}N_4O_3$	568.67
SM4	$C_{27}H_{22}ClN_3O$	439.94

3. Cell viability assay (CCK-8 assay)

To evaluate the effects of candidate compounds on MH7A cell viability and determine appropriate working concentrations for subsequent experiments, a Cell Counting Kit-8 (CCK-8; Dojindo Laboratories, Kumamoto, Japan) assay was performed.

Briefly, MH7A cells were seeded into 96-well plates at a density of 5×10^3 cells/well and cultured for 24 h to allow cell attachment. Cells were then stimulated with TNF-α (20 ng/mL) for 24 h to establish an inflammatory model. Subsequently, the TNF-α-containing medium was removed, and cells were treated with fresh medium containing different concentrations of candidate compounds for an additional 24 h.

The tested concentrations of compounds SM1–SM4 were 3.125, 6.25, 12.5, 25, and 50 μg/mL. After treatment, 10 μL of CCK-8 reagent was added to each well, followed by incubation at 37°C for 2 h in the dark. Absorbance values were measured at 450 nm using a microplate reader.

Cell viability was calculated using the following equation:

$$\text{Cell viability (\%)} = \frac{OD_{sample} - OD_{blank}}{OD_{vehicle} - OD_{blank}} \times 100$$

where OD_{sample} represents the absorbance of compound-treated wells, $OD_{vehicle}$ represents the absorbance of vehicle control wells, and OD_{blank} represents the absorbance of blank wells without cells.

4. Experimental grouping and therapeutic treatment

A therapeutic intervention model was applied to evaluate the anti-inflammatory effects of candidate compounds after inflammatory activation.

Briefly, MH7A cells were first stimulated with TNF-α (20 ng/mL) for 24 h to induce inflammatory responses. Subsequently, TNF-α-containing medium was replaced with fresh medium containing the corresponding candidate compounds, and cells were further cultured for 24 h.

The experimental groups included:

1. Normal control group (NC);
2. Model control group (MD);
3. Vehicle group (Vehicle, model cells treated with the corresponding concentration of DMSO);
4. Candidate compound treatment groups (SM1–SM4).

The NC group received neither TNF-α stimulation nor compound treatment. The MD group was treated with TNF-α only. The Vehicle group was treated with TNF-α and the same concentration of DMSO used as the solvent for the candidate compounds. Candidate compound groups received the corresponding concentrations of compounds SM1–SM4 following TNF-α stimulation.

Based on the CCK-8 results and to minimize potential cytotoxic effects, compounds SM1, SM3, and SM4 were used at 12.5 μg/mL, whereas SM2 was used at 6.25 μg/mL for subsequent inflammatory factor and *C3AR1* expression analyses.

5. Quantitative real-time PCR (RT-qPCR) analysis of inflammatory factors and *C3AR1* mRNA expression

To evaluate the effects of candidate compounds on TNF-α-induced inflammatory responses and *C3AR1* expression, quantitative real-time PCR (RT-qPCR) was performed to measure the mRNA expression levels of *IL6*, *MMP3*, and *C3AR1*.

After compound treatment, total RNA was extracted from MH7A cells using the FastPure® Cell/Tissue Total RNA Isolation Kit V2 (Vazyme Biotech Co., Ltd., Nanjing, China) according to the manufacturer's instructions. RNA concentration and purity were determined before reverse transcription. A total of 200 ng RNA from each sample was reverse transcribed into complementary DNA (cDNA) using the HiScript IV All-in-One Ultra RT SuperMix for qPCR kit (Vazyme Biotech Co., Ltd., Nanjing, China).

RT-qPCR was performed using the SYBR Green-based method with a 2× SYBR Green Master Mix (Vazyme Biotech Co., Ltd., Nanjing, China) in a total reaction volume of 10 μL. Each reaction contained 5.0 μL of 2× Master Mix, 0.2 μL of forward primer, 0.2 μL of reverse primer, 1.0 μL of cDNA template, and 3.6 μL of nuclease-free water. The amplification conditions were as follows: initial denaturation at 95°C for 30 s, followed by 40 cycles of 95°C for 10 s and 60°C for 30 s.

The primer sequences used in this study were synthesized by Sangon Biotech (Shanghai) Co., Ltd. (Shanghai, China) and are listed in Table S2. The expression levels of target genes were normalized to the reference genes *GAPDH* and *ACTB*. Relative gene expression was calculated using the $2^{-\Delta\Delta Ct}$ method.

Table S2. Primer sequences used for RT-qPCR

Gene	Primer set	Forward primer (5′–3′)	Reverse primer (5′–3′)
GAPDH	-	GGAGCGAGATCCCTCCAAAAT	GGCTGTTGTCATACTTCTCATGG
ACTB	-	CATGTACGTTGCTATCCAGGC	CTCCTTAATGTCACGCACGAT
IL6	-	ACTCACCTCTTCAGAACGAATTG	CCATCTTTGGAAGGTTCAGGTTG
MMP3	-	CTGGACTCCGACACTCTGGA	CAGGAAAGGTTCTGAAGTGACC
C3AR1	Set 1	CCCTACGGCAGGTTCCTATG	GACAGCGATCCAGGCTAATGG
C3AR1	Set 2	AAGCCAATCTGGTGTCAGAATC	CAGGAATGCACATCACAAAAGC

C3AR1 mRNA expression was determined using two independent primer sets.

6. Western blot analysis of *C3AR1* protein expression

To further investigate the regulatory effects of candidate compounds on C3AR1 protein expression, Western blot analysis was performed.

After compound treatment, cells were washed twice with pre-cold phosphate-buffered saline (PBS) and lysed in NP-40 lysis buffer containing protease inhibitors on ice for 30 min. Cell lysates were centrifuged at 12,000 × g for 15 min at 4°C, and the supernatants were collected for subsequent protein analysis. NP-40 lysis buffer and protease inhibitors were obtained from Shanghai Panchao Biotechnology Co., Ltd. (Shanghai, China).

Protein concentrations were determined using a bicinchoninic acid (BCA) assay kit (Shanghai Panchao Biotechnology Co., Ltd., Shanghai, China). Equal amounts of total protein (12.5 μg per lane) were separated by 10% SDS-PAGE and subsequently transferred onto polyvinylidene fluoride (PVDF) membranes (Shanghai Panchao Biotechnology Co., Ltd., Shanghai, China) using wet transfer.

The membranes were blocked with 5% skim milk in TBST for 2 h at room temperature and incubated overnight at 4°C with primary antibodies against human *C3AR1* (rabbit polyclonal antibody, 1:5000; ImmunoWay Biotechnology Company) and *GAPDH* (mouse monoclonal antibody, 1:5000; ImmunoWay Biotechnology Company).

After washing, the membranes incubated with C3AR1 primary antibody were treated with HRP-conjugated goat anti-rabbit IgG (H+L) secondary antibody (ImmunoWay) for 2 h at room temperature. Protein bands were visualized using an enhanced chemiluminescence (ECL) reagent (Shanghai Panchao Biotechnology Co., Ltd., Shanghai, China) and quantified by densitometric analysis. C3AR1 protein expression was normalized to GAPDH.

7. Statistical analysis

All experimental data are presented as mean ± standard deviation (SD). Statistical analyses were performed using GraphPad Prism 9.0. Comparisons among multiple groups were conducted using one-way analysis of variance (ANOVA), followed by Tukey's multiple comparison test, where applicable. Different lowercase letters indicate statistically significant differences among groups. A P value < 0.05 was considered statistically significant.
