# Supplementary material for: A Pathway-Driven Computational Drug Discovery Framework Integrating Machine Learning and Virtual Screening for Identification of Anti-Rheumatoid Arthritis Natural Small Molecules
Source: Pharmaceuticals (Basel). 2026 Oct 19;19(10):1651. doi: 10.3390/ph19101651 (PMC13708292; doi:10.3390/ph19101651)

P2000N-59809

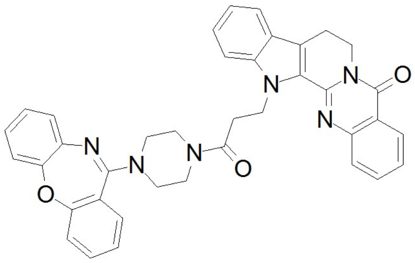

G32852

RRAZNC-01934

$C_{38}H_{32}N_6O_3$ 620.71

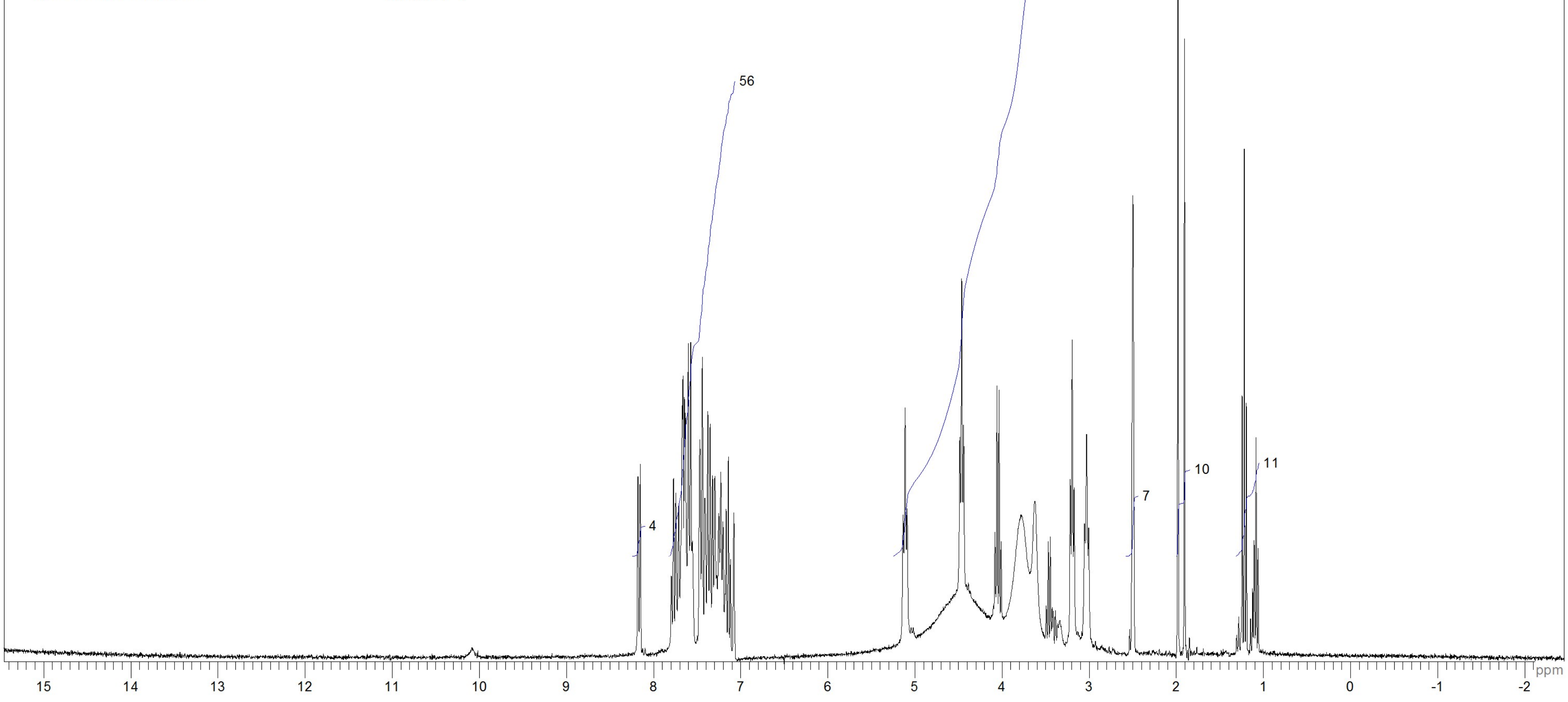

Supplement: Supplementary file 1 [file pharmaceuticals-19-01651-s001.zip › Supplementary_Data_S11/SM1.pdf]
