# Supplementary material for: A Pathway-Driven Computational Drug Discovery Framework Integrating Machine Learning and Virtual Screening for Identification of Anti-Rheumatoid Arthritis Natural Small Molecules
Source: Pharmaceuticals (Basel). 2026 Oct 19;19(10):1651. doi: 10.3390/ph19101651 (PMC13708292; doi:10.3390/ph19101651)
Supplement: Supplementary file 1 [file pharmaceuticals-19-01651-s001.zip › Supplementary_Data_S11/SM2.PDF]

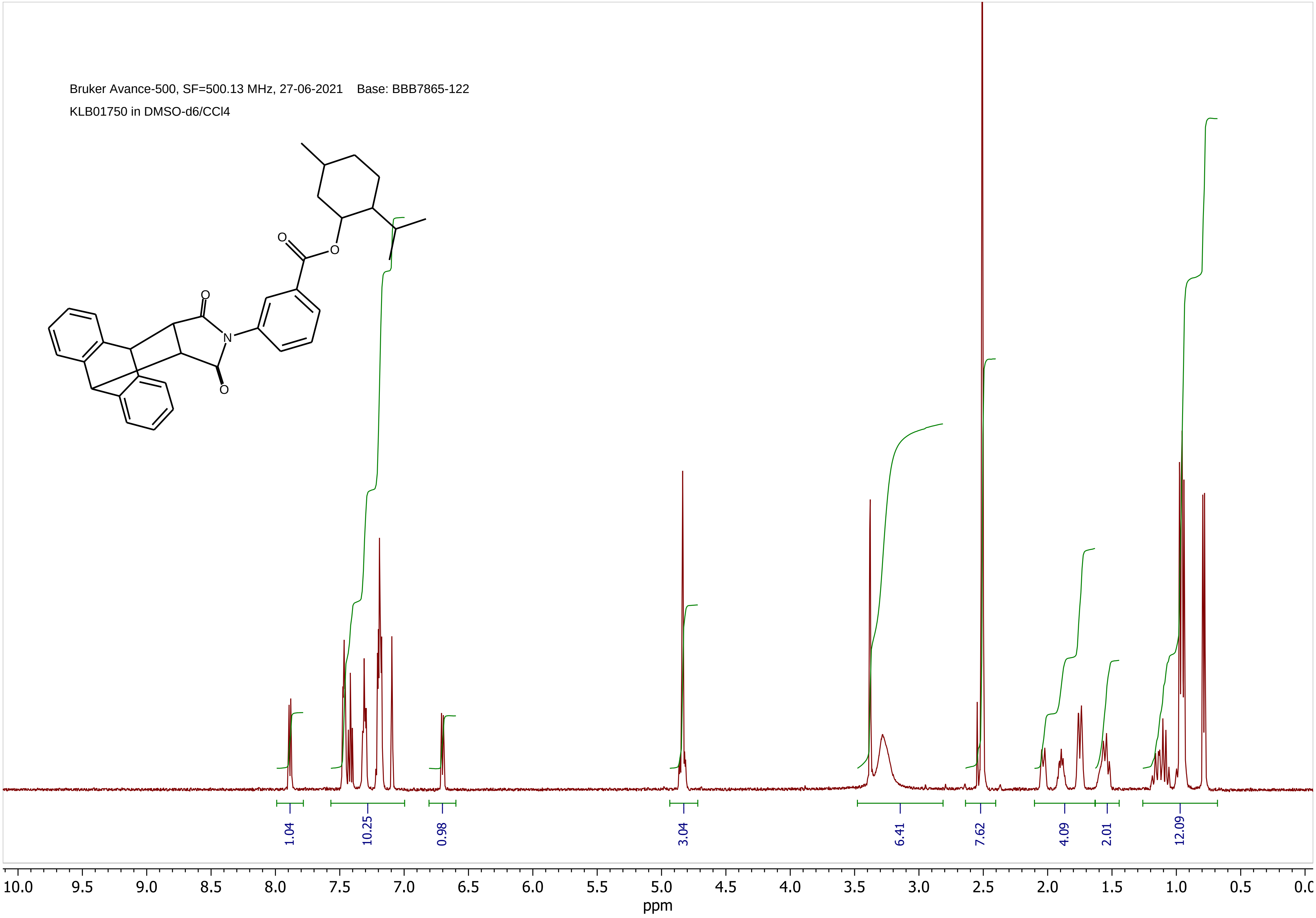

Bruker Avance-500, SF=500.13 MHz, 27-06-2021 Base: BBB7865-122
KLB01750 in DMSO-d6/CCl4
O
O
N
O
O
1.04
10.25
0.98
3.04
6.41
7.62
4.09
2.01
12.09
10.0
9.5
9.0
8.5
8.0
7.5
7.0
6.5
6.0
5.5
5.0
4.5
4.0
3.5
3.0
2.5
2.0
1.5
1.0
0.5
ppm
