# Supplementary material for: A Pathway-Driven Computational Drug Discovery Framework Integrating Machine Learning and Virtual Screening for Identification of Anti-Rheumatoid Arthritis Natural Small Molecules
Source: Pharmaceuticals (Basel). 2026 Oct 19;19(10):1651. doi: 10.3390/ph19101651 (PMC13708292; doi:10.3390/ph19101651)
Supplement: Supplementary file 1 [file pharmaceuticals-19-01651-s001.zip › Supplementary_Data_S11/SM3.pdf]

EX-N0036-0190.nmr

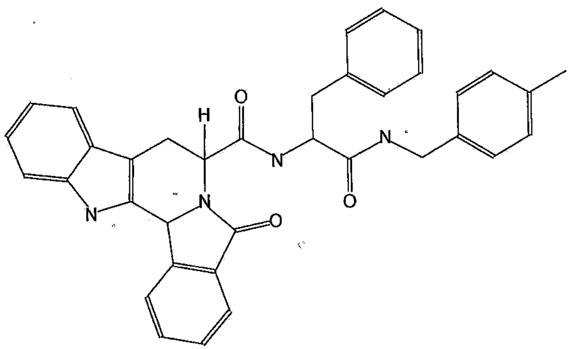

EX-N0036-0190	P2000N-39528

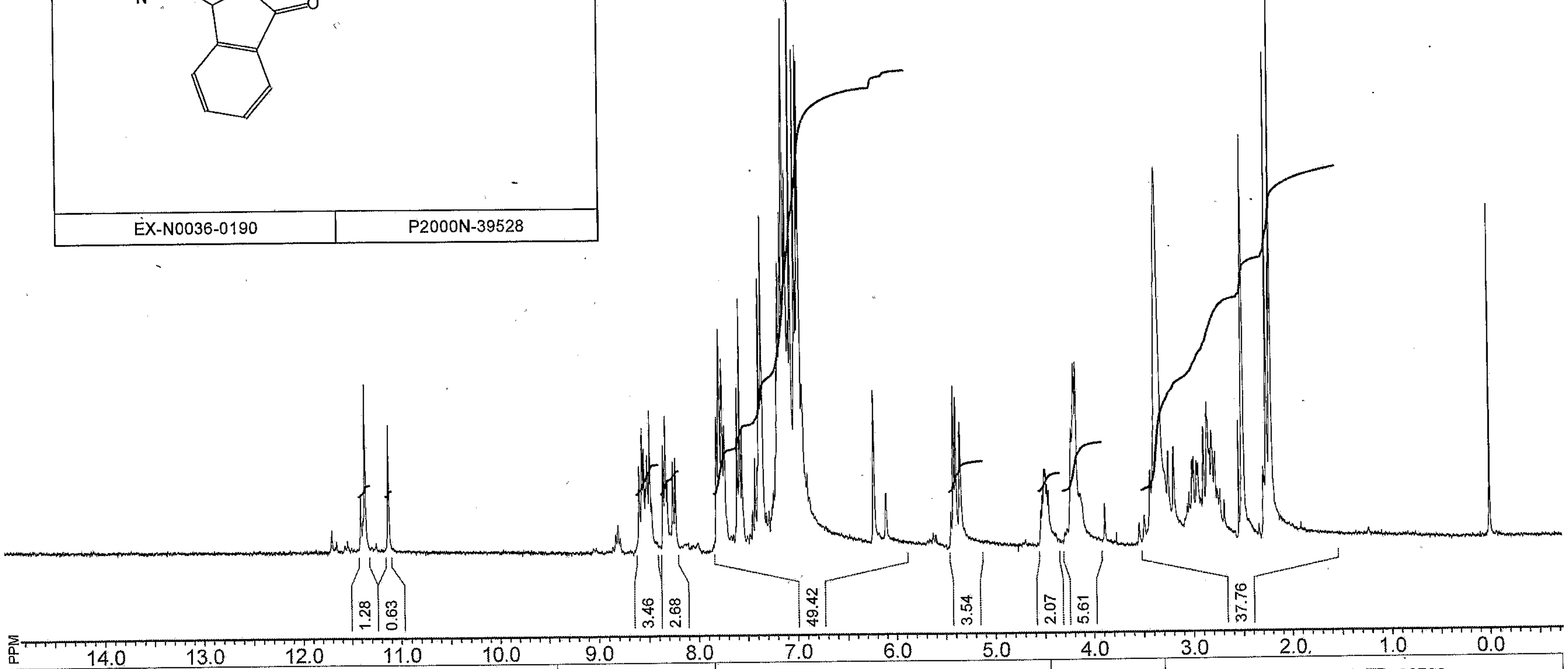

**File name: EX-N0036-0190.nmr**	Owner:	SF: 299.9450 MHz	NS: -1	SI: 16384, TD: 32768
Date: 30-Dec-1899	Solvent:	SW: 5099	TE: 0	SPE in DMSO-D6
