# Supplementary material for: A Pathway-Driven Computational Drug Discovery Framework Integrating Machine Learning and Virtual Screening for Identification of Anti-Rheumatoid Arthritis Natural Small Molecules
Source: Pharmaceuticals (Basel). 2026 Oct 19;19(10):1651. doi: 10.3390/ph19101651 (PMC13708292; doi:10.3390/ph19101651)

Bruker AC-200, SF=200.13 MHz, 02-06-2026    Base: BBB9576-14

IVT15599 in DMSO-d6/CCl4

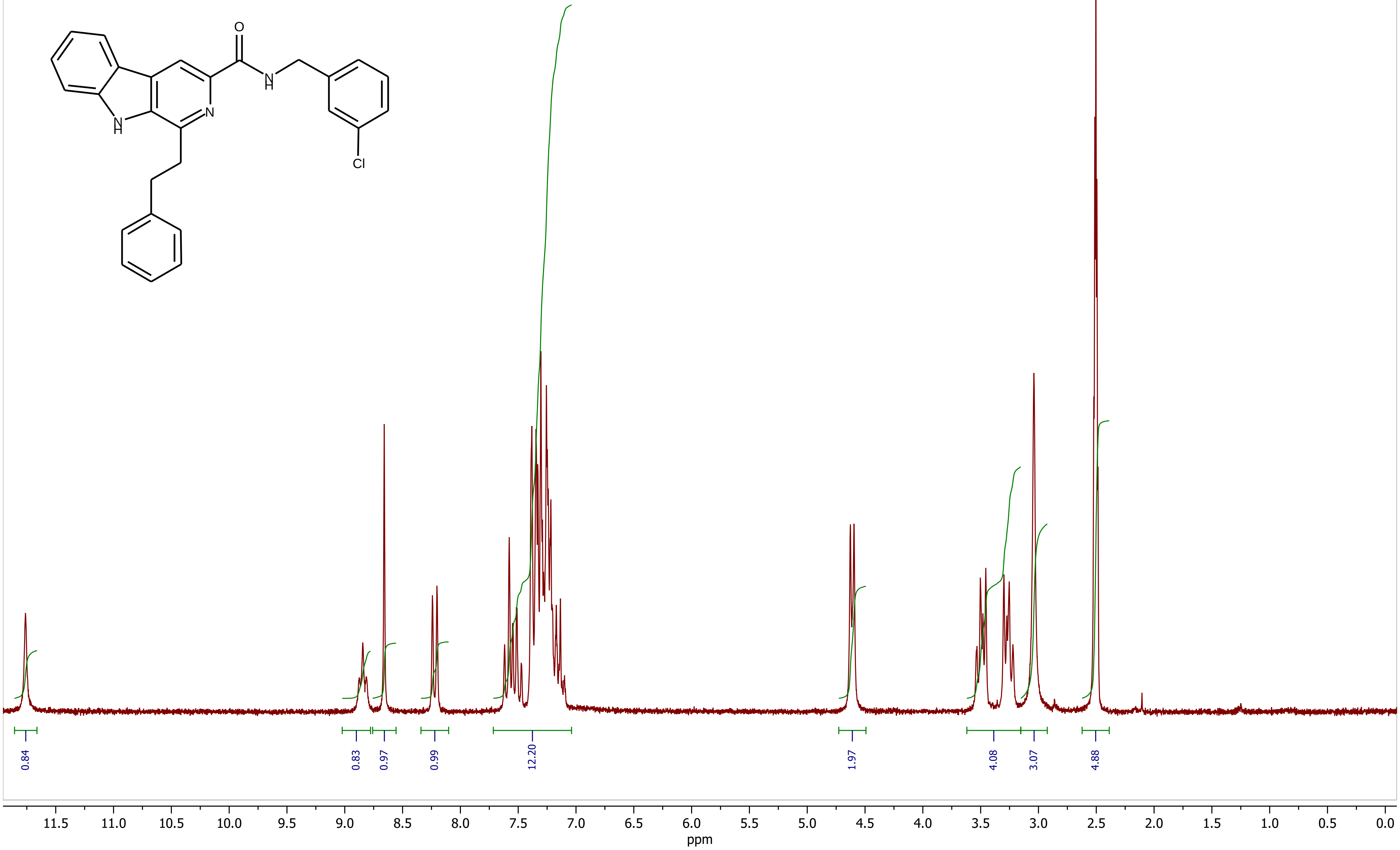

Supplement: Supplementary file 1 [file pharmaceuticals-19-01651-s001.zip › Supplementary_Data_S11/SM4.PDF]
